# An individually adjusted approach for communicating epidemiological results on health and lifestyle to patients

**DOI:** 10.1038/s41598-024-53275-x

**Published:** 2024-02-08

**Authors:** Per Niklas Waaler, Lars Ailo Bongo, Christina Rolandsen, Geir F. Lorem

**Affiliations:** 1 Present Address: Department of Computer Science, UiT The Arctic University of Norway, Tromsø, Norway; 2 Department of Psychology, UiT The Arctic University of Norway, Tromsø, Norway; 3 Deloitte AS, Oslo, Norway

**Keywords:** Risk factors, Rehabilitation, Epidemiology

## Abstract

If scientific research on modifiable risk factors was more accessible to the general population there is a potential to prevent disease and promote health. Mobile applications can automatically combine individual characteristics and statistical models of health to present scientific information as individually tailored visuals, and thus there is untapped potential in incorporating scientific research into apps aimed at promoting healthier lifestyles. As a proof-of-concept, we develop a statistical model of the relationship between Self-rated-health (SRH) and lifestyle-related factors, and a simple app for conveying its effects through a visualisation that sets the individual as the frame of reference. Using data from the 6th (n = 12 981, 53.4% women and 46.6% men) and 7th (n = 21 083, 52.5% women and 47.5% men) iteration of the Tromsø population survey, we fitted a mixed effects linear regression model that models mean SRH as a function of self-reported intensity and frequency of physical activity (PA), BMI, mental health symptoms (HSCL-10), smoking, support from friends, and HbA1c ≥ 6.5%. We adjusted for socioeconomic and demographic factors and comorbidity. We designed a simple proof-of-concept app to register relevant user information, and use the SRH-model to translate the present status of the user into suggestions for lifestyle changes along with predicted health effects. SRH was strongly related to modifiable health factors. The strongest modifiable predictors of SRH were mental health symptoms and PA. The mean adjusted difference in SRH between those with 10-HSCL index = 1.85 (threshold for mental distress) and HSCL-10 = 1 was 0.59 (CI 0.61–0.57). Vigorous physical activity (exercising to exhaustion ≥ 4 days/week relative to sedentary) was associated with an increase on the SRH scale of 0.64 (CI 0.56–0.73). Physical activity intensity and frequency interacted positively, with large PA-volume (frequency ⨯ intensity) being particularly predictive of high SRH. Incorporating statistical models of health into lifestyle apps have great potential for effectively communicating complex health research to a general audience. Such an approach could improve lifestyle apps by helping to make the recommendations more scientifically rigorous and personalised, and offer a more comprehensive overview of lifestyle factors and their importance.

## Introduction

Unhealthy behaviours are related to increased risk of physical illness, mental distress, and reduced health-related quality of life^1–5^. Healthcare services cannot meet the epidemic of lifestyle-related diseases such as cardiovascular disease, obesity, and diabetes type 2, which stresses the importance of facilitating individual adherence to healthy behaviours^6–8^. By following basic health recommendations such as smoking cessation, following a healthy diet, getting sufficient physical exercise, and moderating alcohol consumption, it has been estimated that 70–90% of all lifestyle-related diseases can be treated or prevented, and life expectancy can be increased by more than ten years^9–11^. Although healthy habits are in many cases highly effective at improving health outcomes, most individuals do not adhere to them in the long term without external support or coaching^12–14^. Assisting individuals in developing healthy habits is therefore a central element of nearly all disease rehabilitation and management programs^15–17^. Although the effectiveness of these programs have been well documented, they are not scalable due to resource limitations and the need for long term follow up, as patients tend to relapse into old habits after follow-up is ended^18^. Mobile applications aimed at assisting individuals adhere to a healthier lifestyle, which we refer to collectively as “lifestyle apps”, have been considered as a scalable supplement or alternative to centre-based rehabilitation programmes, and offer several advantages. Lifestyle apps are widely available, can improve communication between healthcare providers and patients, can increase uptake by removing obstacles associated with centre-based rehabilitation sessions (e.g., long travel distance), and provide real-time support and guidance^18–20^. Several studies suggest that lifestyle apps can facilitate effective disease management and long-term adherence to healthy behaviours^21–31^.

Although current lifestyle apps show promise for improving adherence, they currently have three important limitations. Firstly, to the best of our knowledge, they do not provide guidance that accounts for individual characteristics, such as age, mental health status, and sex^32^. This is a missed opportunity, as the optimal strategy for pursuing health depends on individual characteristics, and a personalised experience has been shown to be an important factor for long-term adherence^14^. Using an app as a vehicle for delivering advice and information has the advantage that the information presented can be automatically adjusted based on the user's profile, thus providing a more personalised experience and more relevant recommendations than is possible through generic means of communication, such as standardised guidelines. Secondly, current lifestyle apps typically focus on one aspect of lifestyle in isolation, such as physical activity (PA), weight loss, or dietary changes. Considering modifiable factors in isolation misses the strategically important step of comparing the cost-to-reward ratios (benefit to health and wellbeing relative to investment in terms of personal resources) of various lifestyle changes, and how they might synergise. Finally, current lifestyle apps tend to lack a proper scientific basis^33^, and thus miss an opportunity to utilise the vast amounts of research on the relationship between lifestyle factors and health outcomes which could lead to more effective strategies for improving health if incorporated.

The integration of health research into apps is technically quite feasible, as it is often summarised by statistical models that map individual characteristics to numerical predictions on health outcomes, and this standardised mathematical format conveniently summarises scientific knowledge in a way that can be integrated with relative ease into the automated framework of lifestyle apps. Various measures of health can be used to quantify health outcomes and weigh the importance of lifestyle factors. These can be either objective endpoints, such as life expectancy, or subjective outcomes obtained through self assessment, such as self-rated health (SRH). SRH, a simple one-item instrument that asks a person to rate their health, has multiple advantages in this context. It is easy to collect digitally which allows the user to track their progress over time. Furthermore, it accounts for both subjective and objective aspects of health, and reflects a comprehensive notion of health that includes the positive end of the health spectrum and goes beyond the absence of clinical illness. The fact that it captures health differentials even between clinically healthy individuals means that the sample sizes and timescales needed to study the health effects of lifestyle factors need not be that extreme. SRH is a well validated construct which has been shown to have cross-cultural validity and is a strong independent predictor of mortality even after controlling for a broad range of illnesses and lifestyle factors^34–37^. Thus, SRH offers a suitable way to quantify health for the purpose of integrating health research into a lifestyle app.

We will argue in this article that the aforementioned shortcomings of current lifestyle apps can be efficiently improved upon by integrating research-based statistical models of health into lifestyle apps to form a scientific knowledge base for generating individually tailored recommendations and estimated health benefits for lifestyle changes. Our primary aim is to provide a proof-of-concept that illustrates the advantages and feasibility of this approach by 1. modelling the relationship between SRH and lifestyle factors in a general population, and 2. developing a simple proof-of-concept app that conveys the findings of the research model in terms of health benefits associated with various relevant goals. We aim to highlight how such an approach could improve lifestyle apps by 1. Individualising user feedback in lifestyle apps, 2. Offering a more strategic starting point for lifestyle change by providing a comprehensive overview covering multiple domains of lifestyle change, and 3. Provide a more accurate and precise map of the associations between lifestyle changes and health by incorporating empirical research and mathematical modelling.

To develop the knowledge base for the app we fit a mixed-effects regression model to population data from the 6th and 7th iteration of the Tromsø study, a large-scale population survey with high response rate that draws representative samples from the municipality of Tromsø, Norway, approximately every 7 years. The lifestyle factors considered are self-reported intensity and frequency of PA, body mass index (BMI), smoking, control of blood sugar levels (HbA1c < 6.5%), and symptoms of psychological distress.

## Methods

### Overview of the Tromsø study cohort

To create a knowledge base for the lifestyle recommendation app, we model the associations between lifestyle dependent factors and subjective health using data from the Tromsø population surveys. The Tromsø study is a cohort study initiated in 1974 that invites large representative samples of the municipality of Tromsø, Norway^38^. To achieve higher statistical power in our analysis we model data from the last two iterations, Tromsø6 (2007–08, n = 12 981, 53.4% women and 46.6% men) and Tromsø7 (2015–16, n = 21 083, 52.5% women and 47.5% men). Earlier iterations were not included in our analysis as they used different questionnaire items to measure PA, and the PA-questionnaire items in the last two waves allowed us to analyse the interaction and joint effect of PA frequency and intensity. Protocols for participant sampling were designed with the aim to collect longitudinal data and ensure sufficiently large sample-sizes within different age and gender cohorts^39,40^. In Tromsø6, a 10% random sample of individuals aged 30–39 years, a 40% random sample of individuals aged 43–59, and everyone aged 60–87 years were invited. 65.7% of the invitees of Tromsø6 participated, and the ages ranged from 30 to 87 years. In the Tromsø7 study, all residents of Tromsø aged ≥ 40 years were invited, of which 65.0% participated. The ages of participants ranged from 40 to 99 years. In both studies questionnaires were sent to the participants by email, and physical examinations were carried out for those who chose to physically attend the study.

### Measurements and variables used for modelling

The independent variable of the statistical model we use as a knowledge base in the app is SRH, which is a person's response to the question “How do you, in general, consider your health to be?” with possible answers being 1. Very poor, 2. Poor, 3. Not so good, 4. Good, 5. Excellent. The “very poor” category had negligible responses (0.37% response rate, likely due to the difficulty for those with severe health problems to participate in surveys), and was therefore merged into the “poor” category. After merging, the categories were 1. Poor, 2. Not so good, 3. Good, 4. Excellent. Although SRH only takes discrete values, it reflects underlying states and processes which are continuous, which motivated us to model it as a continuous normally distributed variable. The SRH-predictions can therefore take intermediary values; e.g., if the model predicts a mean difference in SRH of 0.9 associated with a given difference (such as age = 45 and age = 55), then the model predicts that these groups differ on average by almost one SRH level, corresponding approximately to the difference in health between “1. Poor” and “2. Not so good”, or “2. Not so good” and “3. Good”. The modifiable predictors of SRH considered are the following: physical activity (PA) frequency and intensity, body mass index (BMI, categorised into underweight, normal weight, overweight and obese using cut-off values 18.5, 25, and 30 kg/m^2^), mental health symptoms (10 item version of Hopkins symptoms checklist, HSCL), high blood sugar levels (HbA1c ≥ 6.5%), and smoking status (Do you smoke currently? yes/no). As confounders, we included age, sex, education level (1. Primary/partly secondary education [Up to 10 years of schooling], 2. Upper secondary education [a minimum of 3 years], 3. Or having attended college/university), social support (do you have enough friends who can give you help and support when you need it?), household status (do you live with a partner/spouse?), and comorbid disease burden. We modelled only those with data on all model variables.

*Comorbid disease burden* was measured using the health impact index (HII) proposed by Lorem et al.^41^**,** which considers both the joint effect and severity of 11 illnesses, such as Cerebrovascular stroke, Migraine, Myocardial infarction, and Asthma. The presence or history of a condition is measured with questionnaire items of the form “Do you have or have you had …?”. The index is a weighted sum where each term represents the impact on SRH of a medical condition, and the weights have been calibrated based on their association with SRH. For example, the HII of someone who has had a Myocardial infarction (weight = 2) and suffers or has suffered from migraines (weight = 1) is 3. The scale ranges from 0 to 22. HII ≥ 3 is defined as being “seriously ill”^41^.

*PA* levels were measured using self-reported PA frequency (How often do you exercise?) and intensity (If you exercise—how hard do you exercise?). The PA frequency item had responses 1. Never, 2. Less than once a week, 3. Once a week, 4. 2–3 times a week, 5. Approximately every day. The PA intensity item had responses 1. Easy—you do not become short-winded or sweaty, 2. You become short-winded and sweaty, 3. Hard—you become exhausted. We merged the “never” category into the “less than once per week” category to reduce the number of PA frequency categories and simplify the model and analysis. PA frequency and intensity thus had 4 and 3 levels respectively, and there were 12 PA subgroups defined by the combination of PA frequency and intensity.

*Mental health* status was measured using a 10-item version of the HSCL^42^, denoted HSCL-10. HSCL is a well-validated clinical questionnaire for quantifying mental health symptoms that have been developed using factor analysis^43^. Each item has responses 1. No complaint, 2. Little complaint, 3. Pretty much, 4. Very much. The numerical values corresponding to the responses are averaged over the items to produce a summary of the individual's mental health status. Items that lack responses are left out of the calculations, so if 8 items have responses, then the score is the mean over those 8 items. If fewer than seven questions were answered, the HSCL-10 was defined as missing, and the participant's data were excluded from further analysis. HSCL-10 ranges on a scale from 1 to 4, with a high index indicating psychological distress, and an index ≥ 1.85 used as cut-off for mental distress^44^.

### Proof-of-concept app for converting the model to individualised recommendations

**Figure 1:**
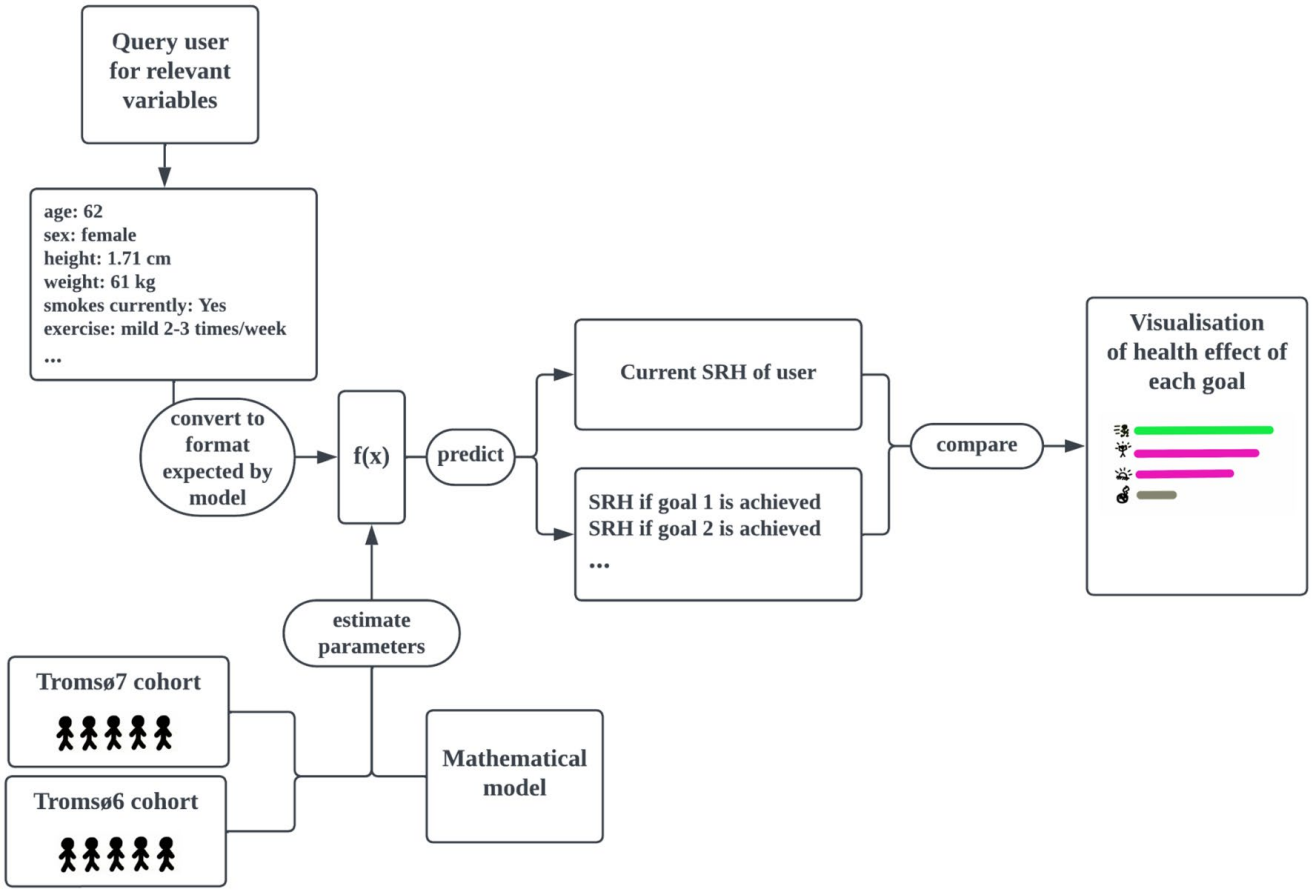
Relationship between individual user, proposed research-based lifestyle app, statistical model, research, and study cohort.

The proof-of-concept app that integrates the SRH-model was developed using Matlab’s app designer. Figure 1 shows a flowchart describing how the app operates and how it relates to research on SRH. It first queries the user for information on the variables needed for the SRH-model to generate predictions using drop-down menus. Once this data is collected, the app identifies modifiable factors for which there is room for improvement. Then, for each such factor (e.g. PA), the estimated effect on SRH of reaching the related goals (e.g. moderate exercise 1–3 times per week) is calculated using the SRH-model. Finally, the goals are presented jointly in a visualisation with descriptive text for each goal placed next to a horizontal bar that indicates the estimated improvement to subjective health if it is achieved. The order of the goals is determined by sorting the goals into categories (exercise, weight loss, blood-sugar control, etc..), and then the categories are ordered by impact of reaching the optimal goal or state (in terms of high SRH) for each respective category, such as a BMI in the normal range. The health effects of some goals, like those related to PA, depend on the degree of change, and we chose, for simplicity, to present only a subset of goals to represent that lifestyle factor. In these cases, we picked goals that seemed relevant for motivating the user, but always include the effect of reaching the theoretically optimal state to show the long-term potential room for improvement.

The app is open sourced under the The GNU Affero General Public License and it is available at https://github.com/uit-hdl/health-diary-app. Collected user data is stored in local files.

### Statistical methods

#### Stratified analyses

We first perform a set of stratified analyses where the data is subdivided into disjoint groups and we calculate various summary statistics to provide a broad empirical overview of the datasets. Sample characteristics (number of participants in various strata and their relative sizes) are collected in Table 1. We calculate the rate of good–excellent SRH in various strata to provide an overview of the associations between the model variables and SRH, which can be seen in Table 2. To empirically assess how the modifiable model variables jointly impact SRH, we stratify the datasets based on the number of unhealthy modifiable factors (UMFs), and calculate the rate of poor or not so good SRH within each UMF strata. The UMFs are defined as high blood-sugar (HbA1c ≥ 6.5%), overweight or obesity, being sedentary, current smoking, or symptoms of mental distress (HSCL-10 ≥ 1.85). The strata consist of those with 0, 1, 2, 3 and ≥ 4 UMF respectively. The results of this analysis are collected in Table 3.

**Table 1:** Sample characteristics for the 6th and 7th wave of the Tromsø survey.

Variable	N.o. cases in Tromsø6 (%)	N.o cases in Tromsø7 (%)
Total number of participants	12,981	21,083
Age ≥ 45	10,107 (77.9%)	17,932 (85.1%)
Age ≥ 55	7698 (59.3%)	11,512 (54.6%)
Age ≥ 65	4017 (30.9%)	5876 (27.9%)
Age ≥ 75	1295 (10.0%)	1728 (8.2%)
Female	6928 (53.4%)	11,074 (52.5%)
Male	6053 (46.6%)	10,009 (47.5%)
Education		
Upper secondary education	3673 (28.7%)	4796 (23.2%)
High school	4289 (33.5%)	5756 (27.8%)
University education (attended)	4836 (37.8%)	10,153 (49.0%)
Comorbid disease burden		
HII = 0	8378 (64.5%)	14,884 (70.6%)
HII ≥ 1	4603 (35.5%)	6199 (29.4%)
HII ≥ 2	3185 (24.5%)	4847 (23.0%)
HII ≥ 3	1430 (11.0%)	1105 (5.2%)
HII ≥ 4	498 (3.8%)	470 (2.2%)
HII ≥ 5	277 (2.1%)	172 (0.8%)
PA frequency		
PA < 1 time/week	2815 (22.3%)	2463 (11.9%)
PA 1 time/week	2504 (19.9%)	3925 (19.0%)
PA 2–3 times/week	4875 (38.7%)	8588 (41.5%)
PA ≥ 4 times/week	2419 (19.2%)	5730 (27.7%)
PA intensity		
PA intensity mild	5473 (47.2%)	7626 (39.1%)
PA intensity moderate	5746 (49.6%)	11,077 (56.7%)
PA intensity hard	377 (3.3%)	824 (4.2%)
BMI		
Underweight (< 18 kg/m^2^)	84 (0.6%)	118 (0.6%)
Normal (18 ≤ BMI < 25 kg/m^2^)	4497 (34.7%)	6720 (32.0%)
Overweight (25 ≤ BMI < 30 kg/m^2^)	5738 (44.3%)	9177 (43.7%)
Obese (BMI ≥ 30 kg/m^2^)	2642 (20.4%)	5005 (23.8%)
Mental health symptoms		
HSCL-10 < 1.85	11,311 (87.1%)	18,633 (88.4%)
HSCL-10 ≥ 1.85 (mental distress)	1090 (8.4%)	1829 (8.68%)
HSCL-10 ≥ 3	75 (0.578%)	126 (0.598%)
Social		
Lives with spouse	9431 (75.1%)	15,283 (76.8%)
Sufficient support from friends	11,014 (88.9%)	18,460 (89.3%)
Smoking		
Smokes currently	2610 (20.4%)	2904 (13.9%)
Control of blood sugar levels		
HbA1c ≥ 6.5%	865 (6.8%)	1238 (5.9%)
Self-rated health		
Poor SRH*	1163 (5.6%)	699 (5.4%)
Not so good SRH	5450 (26.1%)	3699 (28.8%)
Good SRH	11,231 (53.7%)	6592 (51.2%)
Excellent SRH	3059 (14.6%)	1873 (14.6%)

*HSCL* Hopkins symptoms checklist, *PA* physical activity, *HII* Health impact index, *SRH* Self-rated health.

*The category “poor SRH” consists of both “very poor SRH” and “poor SRH”. Percentages are calculated with respect to those who had valid data on the variabel. Health impact index (HII) is a measure of overall comorbid disease burden that weights various medical conditions based on their impact on subjective health.

**Table 2:** Percentage of Tromsø6 and Tromsø7 cohorts experiencing good or excellent Self-rated health within various strata.

Strata	Percentage with good or excellent SRH in Tromsø6 [CI]	Percentage with good or excellent SRH in Tromsø7 [CI]
Age < 65y	68.6% (69.5–70.5)	69.9% (70.6–71.3)
Age ≥ 65y	54.0% (55.5–57.1)	59.2% (60.5–61.7)
Women	62.9% (64.0–65.1)	66.3% (67.1–68.0)
Men	65.4% (66.6–67.8)	67.6% (68.5–69.4)
Education		
Primary/partly secondary	49.8% (51.4–53.0)	52.3% (53.7–55.1)
Upper secondary	63.4% (64.9–66.3)	63.9% (65.2–66.4)
University (attended)	75.6% (76.8–78.0)	75.8% (76.6–77.4)
Comorbid disease burden		
Low burden (HII < 3)	67.9% (68.8–69.6)	68.7% (69.3–70.0)
High burden (HII ≥ 3)	34.1% (36.6–39.1)	36.6% (39.5–42.3)
PA frequency		
Less than 1 time/week	49.8% (51.6–53.5)	50.6% (52.6–54.6)
1 time per week	63.6% (65.5–67.4)	56.3% (57.8–59.4)
2–3 times/week	69.4% (70.7–72.0)	71.0% (72.0–72.9)
At least 4 times/week	70.8% (72.6–74.4)	74.8% (76.0–77.1)
PA intensity		
Mild (brisk walk)	56.0% (57.4–58.7)	56.7% (57.8–58.9)
Moderate (out of breath or sweaty)	75.4% (76.5–77.6)	75.7% (76.5–77.3)
Hard (become exhausted)	84.2% (87.5–90.9)	84.7% (87.0–89.3)
BMI		
Underweight (< 18 kg/m^2^)	36.7% (47.6–58.5)	40.0% (49.2–58.3)
Normal (18 ≤ BMI < 25 kg/m^2^)	69.8% (71.1–72.4)	75.1% (76.2–77.2)
Overweight (25 ≤ BMI < 30 kg/m^2^)	65.4% (66.6–67.8)	68.8% (69.8–70.7)
Obese (BMI ≥ 30 kg/m^2^)	51.1% (53.0–54.9)	52.5% (53.9–55.3)
Mental health symptoms		
Below threshold (HSCL-10 < 1.85)	69.1% (69.9–70.8)	70.7% (71.3–72.0)
Above threshold (HSCL-10 ≥ 1.85)	24.9% (27.2–29.6)	35.9% (38.1–40.2)
Social		
Does not live with spouse	57.5% (59.2–60.9)	60.6% (62.0–63.4)
Lives with spouse	66.7% (67.6–68.6)	69.5% (70.2–70.9)
Insufficient support from friends	41.3% (43.9–46.5)	44.9% (47.0–49.1)
Sufficient support from friends	67.8% (68.7–69.6)	70.1% (70.8–71.4)
Smoking (currently)		
No	67.0% (67.9–68.8)	69.6% (70.3–70.9)
Yes	54.1% (56.1–58.0)	52.2% (54.1–55.9)
Blood sugar control		
HbA1c < 6.5%	66.0% (66.8–67.7)	68.8% (69.4–70.1)
HbA1c ≥ 6.5%	41.8% (45.1–48.4)	41.0% (43.8–46.5)

95% confidence intervals are shown in the brackets.

*SRH* Self-rated health, *HII* Hopkins symptoms checklist.

**Table 3:** Percentage of participants with low Self-rated health within strata defined by the number of modifiable unhealthy lifestyle factors (cohort: Tromsø7 study, 2015).

Number of unhealthy factors	Percentage with poor or not so good SRH	Number of participants
0	14.5% (13.4–15.5)	4249 (20.2%)
1	26.1% (25.2–27.0)	9642 (45.7%)
2	44.0% (42.5–45.5)	4254 (20.2%)
3	57.9% (55.3–60.6)	1331 (6.3%)
≥ 4	79.6% (75.1–84.0)	318 (1.5%)

Unhealthy factors are defined as PA < 1/week, HbA1c ≥ 6.5%), overweight or obesity, current smoking, or symptoms of mental distress (HSCL-10 ≥ 1.85).

*PA* physical activity, *HSCL* Hopkins symptoms checklist, *SRH* Self-rated health.

Study participation can result in selection bias whereby healthier individuals are more likely to participate than seriously ill individuals, especially in our case where we model only those who chose to physically attend the study. If such sampling bias was present, then we would expect the participants who participated in both waves to have different study characteristics than those who only participated in the first wave. To test if a significant sampling bias is present, we performed a stratified analysis comparing age, sex ratios, HII, HSCL, and SRH, for the individuals who participated in both Tromsø6 and Tromsø7 against those who participated only in Tromsø6. The results are presented in the supplementary materials in Table S2. SRH, HSCL-10, BMI, and percentage female participants were similar for the two subgroups. There were significantly more participants who were seriously ill amongst those who did not return for the Tromsø7 survey: 16.34%, compared to 8.6%. Thus it seems that some degree of participation bias may be present. To account for this, we tested if high comorbid disease burden might modify the effect of lifestyle factors, see the subsection “Interaction effects to test generalisability”.

#### The mixed effects regression model

We aim to model mean SRH as a function of modifiable lifestyle factors. To account for dependency between data points in Tromsø6 and Tromsø7 that came from the same individual, we fitted a mixed-effects regression model with participant ID set as the grouping variable. Mixed effects models are suitable for handling longitudinal self-report data, since they can mitigate the effect of self-report bias by comparing consecutive data points collected from a single person and attributing a consistently high or low level to an individual bias rather than to a lifestyle factor.

Age, HSCL-10 and HII were modelled as continuous variables, and the remaining covariates were modelled as categorical. To select power terms for modelling non-linear relationships, we separately fitted univariate models with powers up to degree 4 and kept the powers with p-values < 0.05. With this method, we obtained a model where age was represented with a second-degree term only, HII was modelled with powers 1 and 2, and HSCL was modelled with powers 1–3.

We are assuming that the SRH categories are evenly spaced out (equidistant), so that we can meaningfully talk about an increase in SRH of e.g. 0.6, and so that a fixed change in the covariate based predictor produces the same change to SRH regardless of where on the SRH scale we are located. If this assumption holds, we expect to see a linear relationship between predictions and actual SRH, and departures from linearity would therefore indicate that the assumption is invalid. We visually inspect if this holds by plotting predicted vs actual SRH along with a linear trendline that models predicted values as a linear function of actual values. Specifically, we compare the trendline against the mean predicted value for each SRH category. The result of this analysis can be seen in Fig. 4. In “Justification for treating SRH as a continuous variable” in the supplementary materials we further motivate our use of treating SRH as a continuous variable and the assumption of equidistance by comparing against a Cumulative link model (see Fig. S2 for comparison of coefficients).

#### Interaction effects to test generalisability

To test if differences in age or sex modified the effect of various factors, we utilised interaction terms, which can reveal if changing a factor X can have different effects within different subgroups. A positive interaction between the group A and the factor X means that a unit increase in X is associated with a larger benefit (or less harm) within group A than outside of group A. The interpretation for a negative interaction is similar, but reversed (association is less beneficial/more harmful). If the p-value of the interaction term is insignificant (> 0.05), we conclude that changes in X have the same effect in each subgroup. Specifically, we created an age ≥ 65y category to see if the association between SRH and PA and BMI changes after age 65, which is a commonly used age threshold in the epidemiological literature. We performed a similar analysis for high comorbid disease burden (HII ≥ 3 and HII ≥ 2 respectively) and sex, to see if these variables influence the effect that PA and BMI have on SRH; see “Investigating interaction effects” in the supplementary materials for more details on this analysis.

#### Model presentation and comparison of lifestyle factors

**Figure 2:**
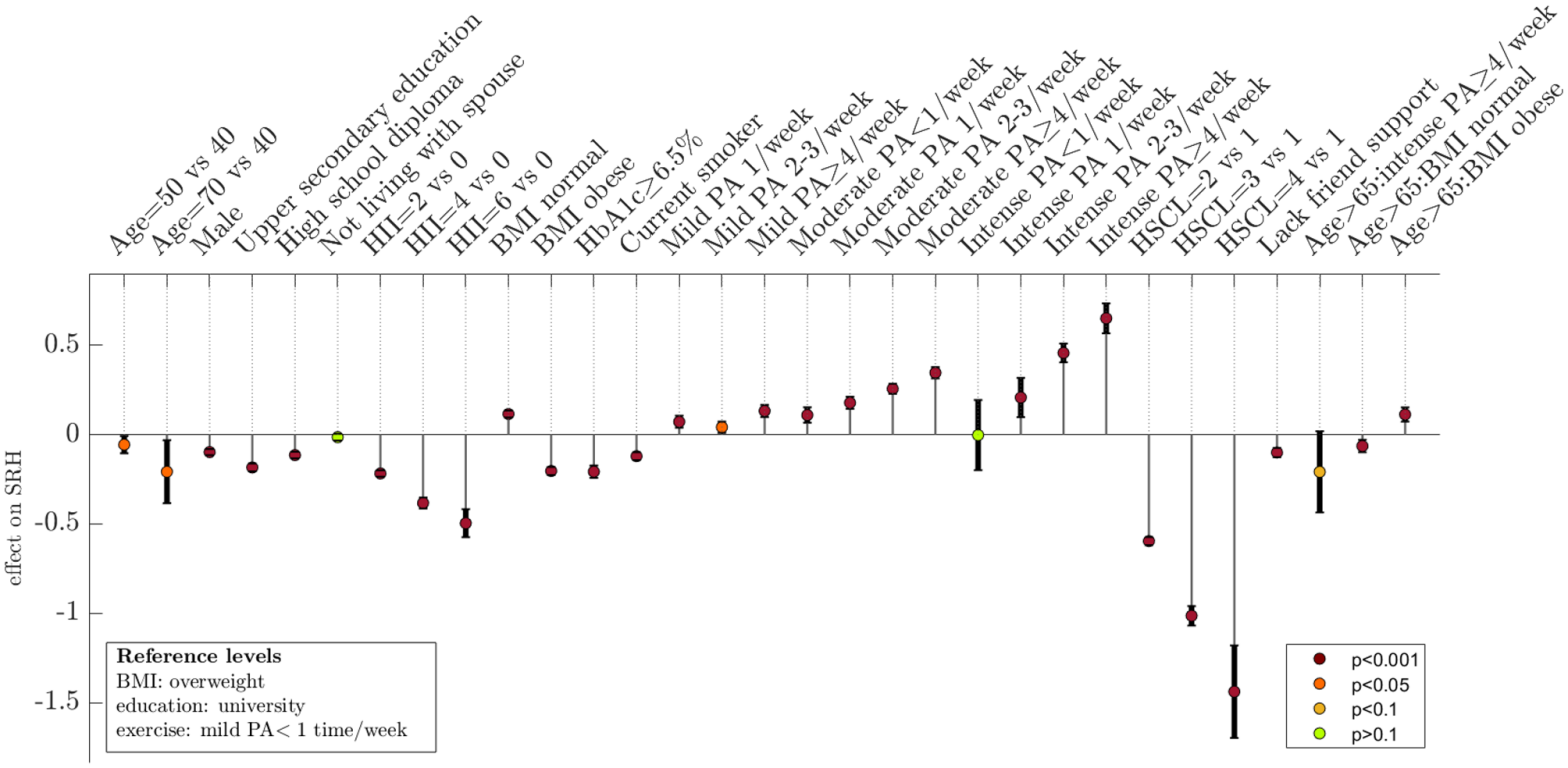
Effects on self-rated health predicted by the multivariate model. To represent the effect of continuous variables (HII, HSCL, and age) we use the SRH-model to estimate effects at selected levels (shown in the figure) compared to the following reference levels: HII = 0, HSCL = 1, and age = 30 years. A unit on the y-axis corresponds to the difference between adjacent levels on a 4-point scale for SRH where the levels range from “1. Poor” to “4. Excellent”. Interaction effects are denoted with “:” (the three bars furthest to the right), and show how the effects of BMI and PA are modified when age > 65y. High non-fasting blood sugar levels are defined as HbA1c ≥ 6.5%. Comorbidity profiles that correspond to HII-levels 2, 4, and 6, respectively are: Heart attack; Heart attack and Cerebrovascular stroke; Heart attack, Cerebrovascular stroke, and Asthma. *HII* Health impact index, *HSCL* Hopkins symptoms checklist, *PA* Physical activity, *SRH* self-rated health.

We present the relationships captured by the model in terms of model-predicted effects; the predicted mean change in SRH when a covariate X is changed from some reference value to a new value, assuming all other covariates are held fixed. To present an intuitive overview of the non-linear relationship between age and SRH, we predict the effect of age increasing from a reference value of 40 years up to 50 and 70 years respectively. The result can be seen in Fig. 2, where we treated HII and HSCL-10 similarly, using reference values HII = 0 and HSCL-10 = 1. For easier comparison of the impact of the most important variables in the model we calculate the effects of changing HII, HSCL-10 and PA-levels from some specified reference level to a new level (note: “change” and “effect” in this paper are used for convenience, and are not meant to imply that an effect is causal or that a variable is modifiable). The effects calculated in this analysis and the specific levels chosen for comparison can be seen in Table 5. Confidence intervals for effects were computed using the Delta method and parameter covariance estimates. Uncertainties are reported with p-values and 95% confidence intervals, with p-values below 0.05 considered significant.

#### Investigating interactions between exercise intensity and frequency

**Figure 3:**
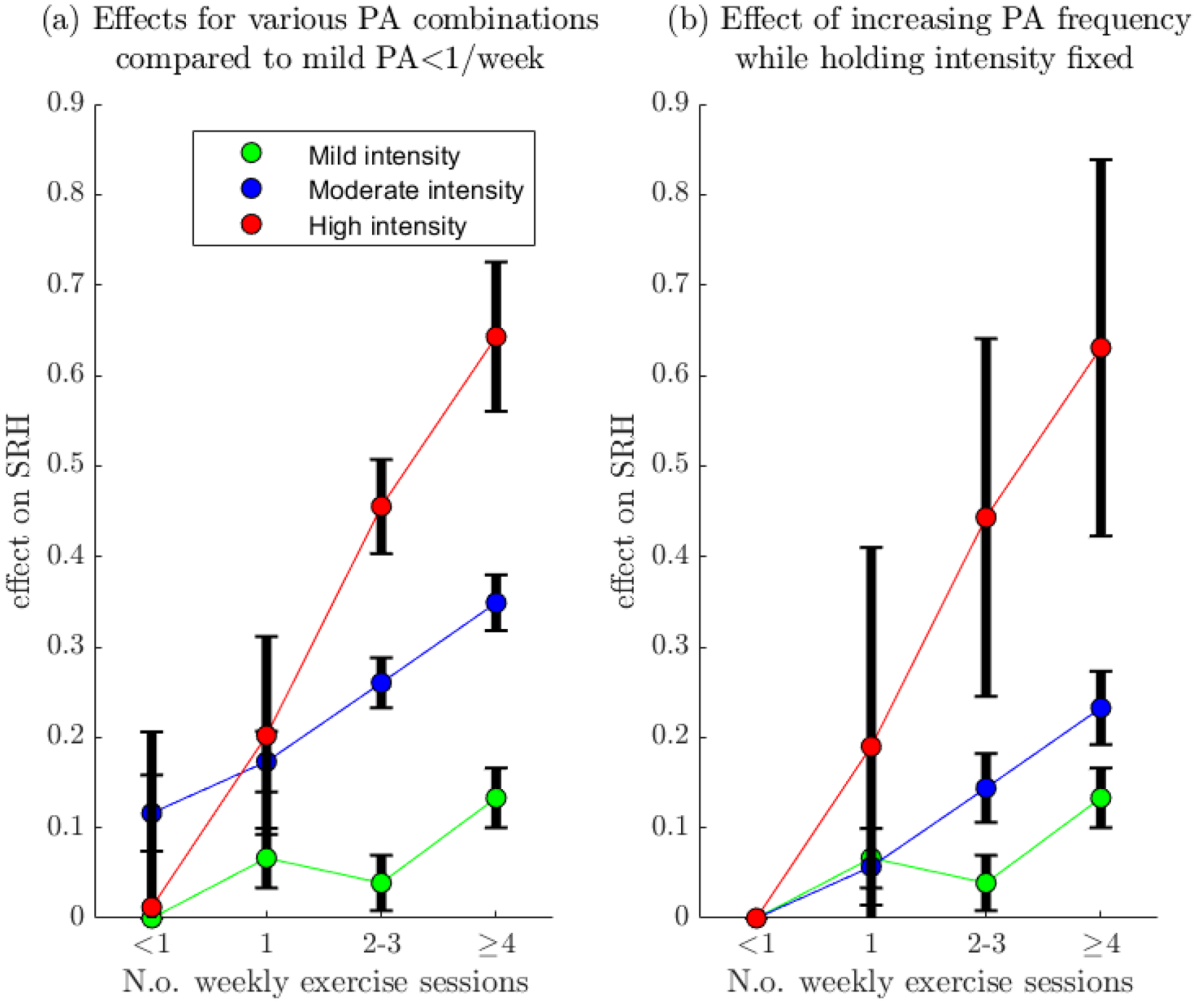
Effect of Physical activity intensity and frequency on self-rated health. Panel (**a**): Effect on SRH of changing PA category from the baseline group: mild intensity < 1/week (the sedentary group). Panel (**b**): Effect on SRH of increasing PA frequency, from “ < 1 time/week” to “ ≥ 4 times/week”, while holding the PA intensity fixed at mild, moderate, and high respectively. For example, the red trajectory shows the effect of exercising with high intensity < 1 time/week, 1 time/week, 2–3 times/week, and ≥ 4 times/week respectively. *PA* Physical activity, *SRH* self-rated health.

Our dataset is well suited for exploring how PA intensity and frequency interact in their effect on SRH, since these variables are separated in Tromsø6 and Tromsø7. An increase in the number of weekly sessions will result in a higher increase in overall PA-volume if the intensity of the PA is high. It is therefore plausible that intensity and frequency will interact positively in their effect on SRH, with larger gains in mean SRH when the intensity is high. To explore if this is the case, we compute the effects of increasing the PA frequency from the baseline of < 1 time/week while holding PA intensity fixed. Interaction effects can then be analysed visually by comparing the slope of the trajectory associated with each intensity level (Fig. 3b). For an overview of the effect of various combinations of PA frequency and intensity, we also compute effects for each such combination with the sedentary group as the reference category (Fig. 3a).

#### Parameter sensitivity to selection of model variables

Several of the model covariates, such as PA, BMI, mental distress, and comorbid disease burden, influence each other through a multitude of different causal pathways. In the case of PA, it can be argued that BMI, comorbid disease burden, and mental health can be viewed as confounders but also as intermediary variables through which PA causally impacts health. A similar relationship can be argued for between HII and HSCL-10 with BMI, and BMI has considerable collinearity with blood-sugar levels. The complicated nature of these relationships makes deciding which variables to include in the model difficult and subjective. If the effects predicted by the model are highly sensitive to these choices, the validity of the model as a basis for recommending lifestyle goals is questionable. We therefore perform a sensitivity analysis where we investigate how the model predictions on the effect of changing PA levels or BMI are influenced by inclusion of these variables. Specifically, for PA, we fit a base model M0 that adjusts for age, education, sex, smoking, and then create a nested sequence of models by adding one variable at a time, thus obtaining: M1 = M0 + BMI, M2 = M1 + HII, and M3 = M2 + HSCL-10. Using Models 1–3, we then calculate the effect PA has on SRH by comparing the difference in mean SRH between “mild PA < 1 time/week” to “hard PA ≥ 4 times/week”. For BMI, we do a similar analysis, adjusting for the same variables in the baseline model, with the sequence of models now being M1 = M0 + HII, M2 = M1 + HSCL-10, and M3 = M2 + HbA1c. The results of this analysis is shown in Fig. S1 in the Supplementary materials**.**

We have modelled SRH as a continuous normally distributed variable, and to assess the validity of this assumption we compare it to a matching discretized normal distribution; specifically, we fit a normal distribution to SRH, use the fitted distribution to compute theoretical rates for each SRH level by computing the probability mass over the bins with endpoints [-∞, 1.5, 2.5, 3.5, ∞]. The goodness of fit was assessed by comparing theoretical (using the fitted normal distribution) and empirical probabilities. The results of this analysis can be seen in Fig. S4 in the supplementary materials. 

#### Assessing model accuracy

To test for overfitting and assess the predictive capabilities of the SRH-model, we set aside a randomly selected test set of 200 participants prior to performing any analysis. To ensure that the train and test set had no participants in common (thus ensuring complete independence), we included in the test set only Tromsø7 participants who had not participated in Tromsø6. Since the SRH-model predicts continuous values but SRH takes discrete values from 1 to 4, we convert the continuous SRH-predictions to discrete value predictions by rounding them to the nearest integers in the range 1–4. As the primary performance metric, we compute the accuracy; the fraction of model predictions that were correct. We compare the prediction accuracy for these metrics between the test set and the set used to fit the model to assess if the model is overfitted to the dataset. We also calculate the area under the receiver operating characteristic curve (AUC) which reflects a models’ potential ability to separate the data into two non-overlapping classes. To test if prediction ability is symmetrical, i.e. if it is equally useful for predicting the positive and negative end of the SRH scale, we calculate the AUC for predicting poor SRH and excellent SRH respectively. To get a sense of how much of the variation in SHR is explained by the model, we calculate the marginal R^2^: the proportion of the total variance explained by the fixed effect coefficients, which ranges from 0 to 1, with 1 indicating that the independent variable can be perfectly predicted from the model covariates^45^. Finally, we report the standard deviation of the random effects (the individual SRH baselines) as an estimate of how much variation there is in reporting behaviour between individuals.

#### Comparing models

It is therefore interesting to consider what performance can be gained by utilising more complex and flexible models and machine learning techniques and shift focus to pure performance optimisation, since presentation via apps simplifies the output equally regardless of model complexity. This also helps us analyse to what degree the model's accuracy is limited by inefficient usage of data or lack of information in the input data. To this end, we compare the performance of the mixed-effects model in fivefold cross validation against two machine learning algorithms that have demonstrated high performance on structured data: Explainable boosting machines (EBM) and Extreme Gradient Boosting (XGboost). We also tested adding data on variables (alcohol consumption and heart rate) that were not included in the main model due to concerns relating to effect interpretation. More details on this comparison can be found in the Supplementary materials.

### Ethics approval and consent to participate

The Regional Ethical Committee of Northern Norway gave ethical approval for this work (project number 89721). The Tromsø study was approved by the Norwegian Data Inspectorate and the Regional Ethical Committee of North Norway (REK). The methods of this study were performed in accordance with the relevant guidelines and regulations. The Tromsø Study collected written informed consent from all participants.

## Results

### Cohort and data characteristics

The number of participants with data on all relevant variables is listed in Table S1. 22 335 participants had complete data in either Tromsø6 or Tromsø7, and data from these participants formed the subset used to model SRH with the mixed effects model. 6 264 of these individuals participated in both Tromsø6 and Tromsø7, and thus provided the longitudinal data that the model could use to estimate random effects. Sample characteristics of the Tromsø6 and Tromsø7 cohorts can be seen in Table 1, which shows that the datasets are broad and offer good representation across many subgroups. Both waves contain a large number of participants also from older age groups, including those aged ≥ 75y (1295 and 1728 respectively), making the dataset suitable for modelling age-dependent effects. Men and women are approximately equally represented. Education level is also fairly evenly distributed across the various levels. The datasets also contain fairly high numbers of individuals with high comorbid disease burden (HII ≥ 3; 1430 [11.0%] and 1105 [5.2%] participants respectively) which allows us to investigate if the model effects generalise to this subgroup. Smoking was the only variable that had a notably different distribution, with the percentage of current smokers dropping from 20.4% in Tromsø6 to 13.9% in Tromsø7.

Table 2 shows the percentage of participants who experience good or excellent SRH within various subgroups, or strata, in Tromsø6 and Tromsø7. Higher education, support from friends, low comorbidity burden, frequent and intense PA, low mental distress, support from friends, and blood sugar levels below the diabetic threshold were associated with substantially increased likelihood (> 20% absolute difference between levels) of good–excellent SRH. 36.6% of those with high comorbidity index (5.2% of Tromsø7) reported good–excellent SRH vs 68.7% for those with HII < 3; an absolute difference of 32.1%. However, the variable associated with the largest inter-group difference was between the high and low mental distress subgroups: 35.9% rate of good–excellent SRH for those with significant mental distress (8.4% of Tromsø7) vs 70.7% for those with HSCL-10 < 1.85 in Tromsø7 (a 34.8% absolute difference). The patterns in the rate of good–excellent SRH across different subgroups appear similar in both surveys, indicating that relationships between SRH and the model variables are reasonably stable across time.

Table 3 shows prevalence of poor or not so good SRH stratified by the number of unhealthy modifiable factors (UMF) in the Tromsø7 cohort. The majority, 65.9%, had only 0–1 unhealthy factors. A clear trend can be seen whereby low SRH is far more likely with higher numbers of UMFs. Those with ≥ 4 UMFs rated their health as poor or not so good at a rate 5.5 times higher than those with 0 UMF, suggesting that SRH is highly highly influenced by modifiable factors. The rate of poor or not so good SRH increases with the number of UMFs by increments (in absolute %) of 11.6%, 17.9%, 13.9% and 21.7%, suggesting that the effects on SRH are approximately additive.

### Model effects

Table 4 shows the model parameters after fitting the mixed effects regression model to Tromsø6 and Tromsø7 data with 95% confidence intervals. The model's marginal R^2^ was 0.631, indicating 63.1% of the variance in SRH is explained by the fixed effects. The standard deviation of the random effects (the individual SRH-baselines) was 0.298, indicating that considerable variability in SRH is due to differences in individual SRH baselines.

**Table 4:** Coefficients of the mixed effects regression model fitted to the Tromsø6 and Tromsø7 surveys.

Variable	Coefficient estimate (95% CI)
Confounders	
Age	0.037 (− 0.024, 0.097)
Age^2	− 0.006* (− 0.011, − 0.00075)
Male	− 0.096*** (− 0.11, − 0.079)
Not living with spouse	− 0.01775 (− 0.040, 0.001)
Lack friend support	− 0.12*** (− 0.14, − 0.091)
Primary/partly secondary education	− 0.18*** (− 0.2, − 0.15)
Upper secondary education	− 0.11*** (− 0.13, − 0.096)
HII	− 0.12*** (− 0.14, − 0.11)
HII^2	0.0069** (0.0029, 0.011)
Modifiable variables	
BMI normal	0.11*** (0.095, 0.13)
BMI obese	− 0.21*** (− 0.23, − 0.18)
Blood sugar control (HbA1c ≥ 6.5%)	− 0.21*** (− 0.24, − 0.17)
Current smoker	− 0.12*** (− 0.14, − 0.099)
Mild PA < 1/week	− 0.26*** (− 0.29, − 0.23)
Mild PA 1/week	− 0.19*** (− 0.22, − 0.17)
Mild PA 2–3/week	− 0.22*** (− 0.25, − 0.2)
Mild PA ≥ 4/week	− 0.13*** (− 0.15, − 0.1)
Moderate PA < 1/week	− 0.15*** (− 0.18, − 0.11)
Moderate PA 1/week	− 0.087*** (− 0.11, − 0.06)
Moderate PA ≥ 4/week	0.089*** (0.066, 0.11)
Intense PA < 1/week	− 0.25** (− 0.44, − 0.056)
Intense PA 1/week	− 0.06 (− 0.17, 0.048)
Intense PA 2–3/week	0.19*** (0.15, 0.24)
Intense PA ≥ 4/week	0.38*** (0.3, 0.46)
HSCL	− 1.9***(− 2.2, − 1.5)
HSCL^2	0.56*** (0.37, 0.75)
HSCL^3	− 0.063*** (− 0.095, − 0.032)
Interaction variables	
Age > 65 : intense PA ≥ 4/week	− 0.24* (− 0.47, − 0.02)
Age > 65 : BMI normal	− 0.073*** (− 0.11, − 0.04)
Age > 65 : BMI obese	0.11*** (0.074, 0.15)

Self-rated health is modelled as a continuous variable, with a unit change representing a one-level increase on the 4-point SHR scale (e.g., from “poor” to “good”). Interaction is denoted with “:” (the bottom three rows). The unit of age is 1 decade. Reference categories are: moderate PA intensity, exercising 2–3 times/week with moderate intensity, overweight BMI, university level education (attended), and age ≤ 65. Significance levels encoding: (***) for p < 0.001; (**) for p < 0.01; (*) for p < 0.05.

*HII* health impact index, *HSCL* Hopkins symptoms checklist, *SRH* self-rated health, *PA* physical activity.

For a visual overview of the direction and magnitude of effects predicted by the multivariate model and the uncertainty of these estimates see Fig. 2, which shows the effects (relative to specified reference levels or groups) as vertical lines with 95% confidence intervals. The strongest predictors of SRH in the adjusted model were HSCL-10, HII, PA, and BMI; see Table 5 for an overview of the effect of the most impactful changes according to the model. Figure 3a shows the mean adjusted SRH for different combinations of PA frequency and intensity compared to the participants who were sedentary, and it is clear that higher PA volume is strongly associated with higher SRH; the combination of PA frequency and intensity associated with the highest SRH was hard PA ≥ 4 times/week (effect = 0.64, comparing hard PA ≥ 4 times/week to sedentary). High PA-volume, low levels of mental distress, and BMI in the normal range predicted high SRH. Both underweight and overweight were associated with lower SRH, though the effect of underweight was not significant. Other predictors associated with higher SRH were female sex, not smoking, high education level, receiving sufficient support from friends, and non-fasting blood sugar levels in the diabetic range (HbA1c < 6.5%). Using the multivariate model to investigate the effect of making multiple changes simultaneously, we estimated that reducing BMI from obese to normal, increasing PA from from the least to most active category, and reducing the HSCL-10 index from level 3 to level 1, was associated with an SRH increase of 1.9, approximately equivalent to a change from “poor” to “good”.

**Table 5:** Effects on SRH predicted by the multivariate SRH model for the most impactful covariates.

Variable type	Change	Effect on mean SRH
Mental health symptoms	From HSCl-10 = 4 to HSCl-10 = 1	1.27
Mental health symptoms	From HSCl-10 = 1.85 to HSCl-10 = 1	0.588
Comorbid disease burden	From HII = 0 to HII = 6	0.42
Physical activity	From “mild < 1/week” to “hard ≥ 4 times/week”	0.643
BMI	From “obese” to “normal”	0.32

The multivariate mixed effects regression model was used to estimate the adjusted effects for making the indicated changes (in the mathematical sense) for the most impactful model variables, to showcase how much each variable can potentially influence SRH. SRH ranges from values 1 to 4, and the largest possible group difference in mean SRH is 3. An example of a comorbidity profile corresponding to HII = 6 is Myocardial infarction, Cerebrovascular stroke, and Migraine (currently or historically). HSCL-10 = 1.85 is defined as the threshold for significant mental health symptoms.

*SRH* Self-rated health, *HII* Health impact index, *HSCL* Hopkins symptoms checklist, *PA* Physical activity.

We observed significant interactions between age ≥ 65y and BMI, indicating that age ≥ 65y modifies the association between BMI and SRH. Based on how the association with SRH was modified, BMI reduction appeared less beneficial, and BMI increase less harmful. For people aged ≥ 65y, the interaction with obese BMI was positive, so an increase in BMI from overweight (the baseline category) to obese appeared less harmful in this age group. Consistent with this trend, the interaction between normal BMI and aged ≥ 65y was negative, indicating that a reduction in BMI from overweight to normal appeared less beneficial for those aged ≥ 65y. Underweight BMI and aged ≥ 65y interaction also followed this trend of, but here the p-value of the interaction effect was not statistically significant after adjusting for mental health. We also observed a negative interaction (-0.253, p = 0.026) between age ≥ 65y and the most vigorous level of exercise (≥ 4 times per week of intense exercise), indicating that this level of PA was not as strongly associated with good SRH as it was for those with age < 65y. PA frequency and intensity interacted positively in their effect on SRH. The effect of PA or BMI on SRH was not modified significantly by either sex or HII, indicating that the effect on SHR of these variables might generalise across stratifications based on sex or HII (see Table S3 for exact p-values and details on this analysis).

A positive interaction was visually observed between PA frequency and PA intensity. Figure 3b shows the model-estimated effect of increasing PA frequency while holding PA intensity fixed, and it is visually clear based on the slopes of the trajectories within each intensity-strata that increases in PA frequency are associated with larger increases in mean SRH within the strata of participants that reported higher exercise intensity. Surprisingly, in the strata of participants who reported exercising with mild intensity, the adjusted mean SRH was lower in the subgroup that exercises 1–3 times/week than in the subgroup that exercised < 1 time /week, despite reporting exercising more often. This paradoxical finding is discussed in the supplementary materials.

### Test set predictions and model performance

**Figure 4:**
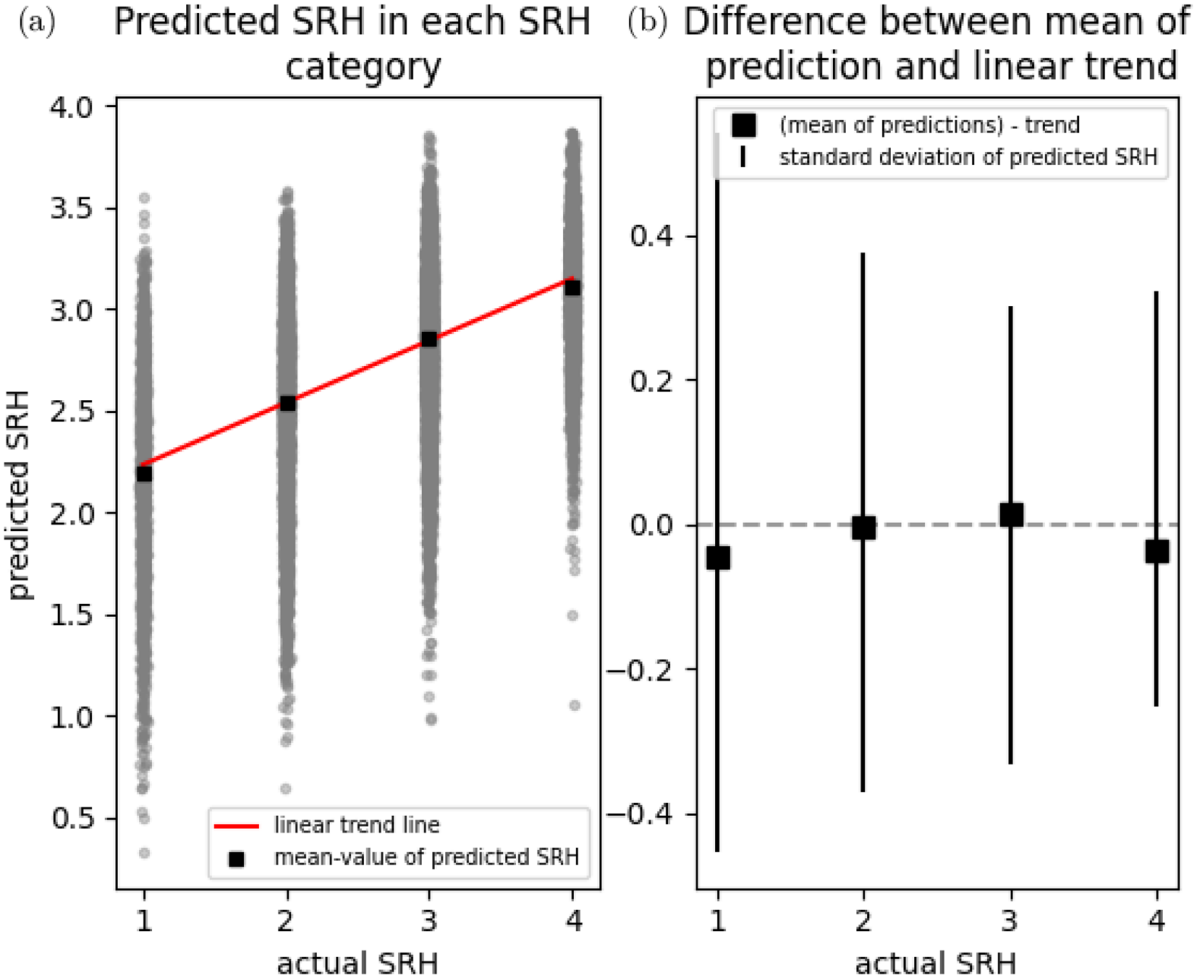
Linearity of the relationship between predicted and actual Self-rated health. Panel (**a**) shows the actual vs the predicted SRH values on the training set, together with a fitted trendline obtained by fitting a linear model with predicted SRH as a function of actual SRH. Panel (**b**) In each SRH category, we subtracted the mean of the predicted values from the corresponding values of the trendline shown in panel (**a**), effectively zooming in on these differences. The differences are plotted along with the standard deviation of the SRH predictions to provide an appropriate scale. Both figures show that the predicted values increase in a near-perfect linear fashion with SRH values. *SRH* self-rated health.

**Figure 5:**
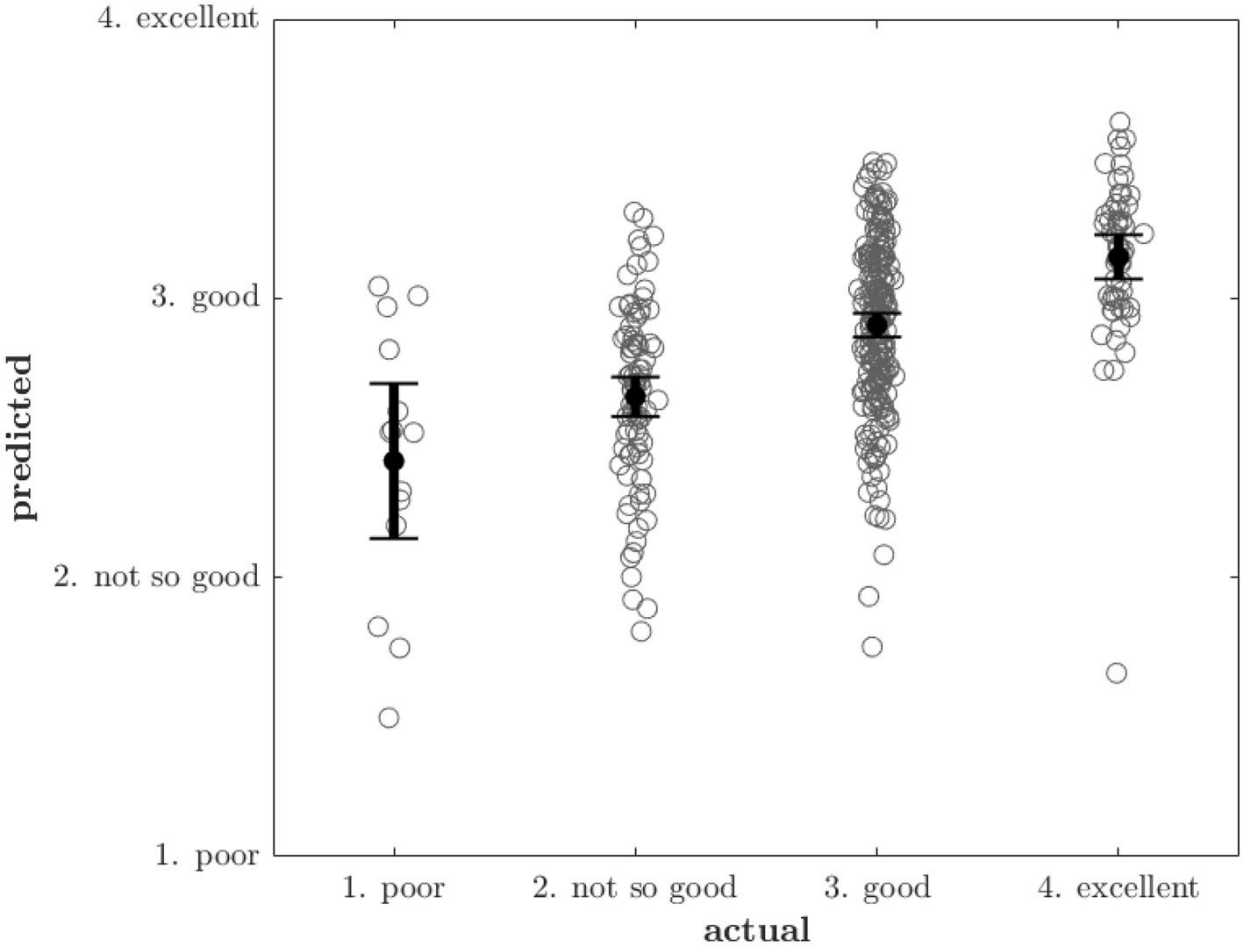
Actual self-rated health values vs Model predictions on the test-set. Small random perturbations have been added to the x-coordinate (actual SRH) in order to better visualise the distribution of the predicted values. For participants in each SRH strata, we computed 95% confidence intervals for the mean of the predicted SRH. *SRH* self-rated health.

Figure 5 shows model predictions of SRH on a test set consisting of 200 participants who participated in Tromsø7 but not Tromsø6. Figure 4 shows similar information for the training set, but also includes a linear trendline for predicted vs true values, and compares this trend to the mean predicted values in each SRH-category. Departures from the linear trend are small, and the fact that the mean of the predicted values appear to grow in a linear fashion with increasing SRH supports the regression models assumption of equidistance between the SRH levels. Figure 4 and 5 also show considerable scatter indicating that the model has limited accuracy. The standard deviation of the random effects (the individual SRH-baselines) was 0.298; this indicates a fairly high variability in baseline SRH between individuals which could explain the model's limited accuracy. Consistent with this finding is that we found negligible differences in cross-validation performance between models when we compared the main model to more flexible models that can potentially make better use of the input data by virtue of their flexibility (see Table S4).

Table 6 shows prediction metrics for the model on the test and training set. The test set prediction metrics give no indication that the model is overfitted. The model has fairly good accuracy for discerning SRH at the extremes, “poor” and “excellent”, with an AUC of ~ 0.8 (training set) in both cases. Figure 5 shows that the absolute predictions tend to be very conservative; this is likely due SRH being an inherently difficult target to predict, which discourages extreme predictions since these are heavily penalised when incorrect. The results indicate that SRH can be predicted with decent accuracy from PA levels, BMI, HSCL-10, comorbid disease burden.

**Table 6:** Model prediction metrics.

Metric	Evaluation on test set	Evaluation on training set
Accuracy predicting SRH (level 1 to 4)	0.53 (0.46–0.60)	0.59 (0.58–0.59)
AUC predicting poor SRH	0.88 (0.68–1.0)	0.80 (0.77–0.81)
AUC predicting excellent SRH	0.79 (0.68–0.90)	0.79 (0.78–0.80)
Correlation with actual SRH	0.59 (0.48–0.68)	0.53 (0.53–0.54)

*SRH* Self-rated health.

### Study based recommendations for fictional users of the lifestyle app

**Figure 6:**
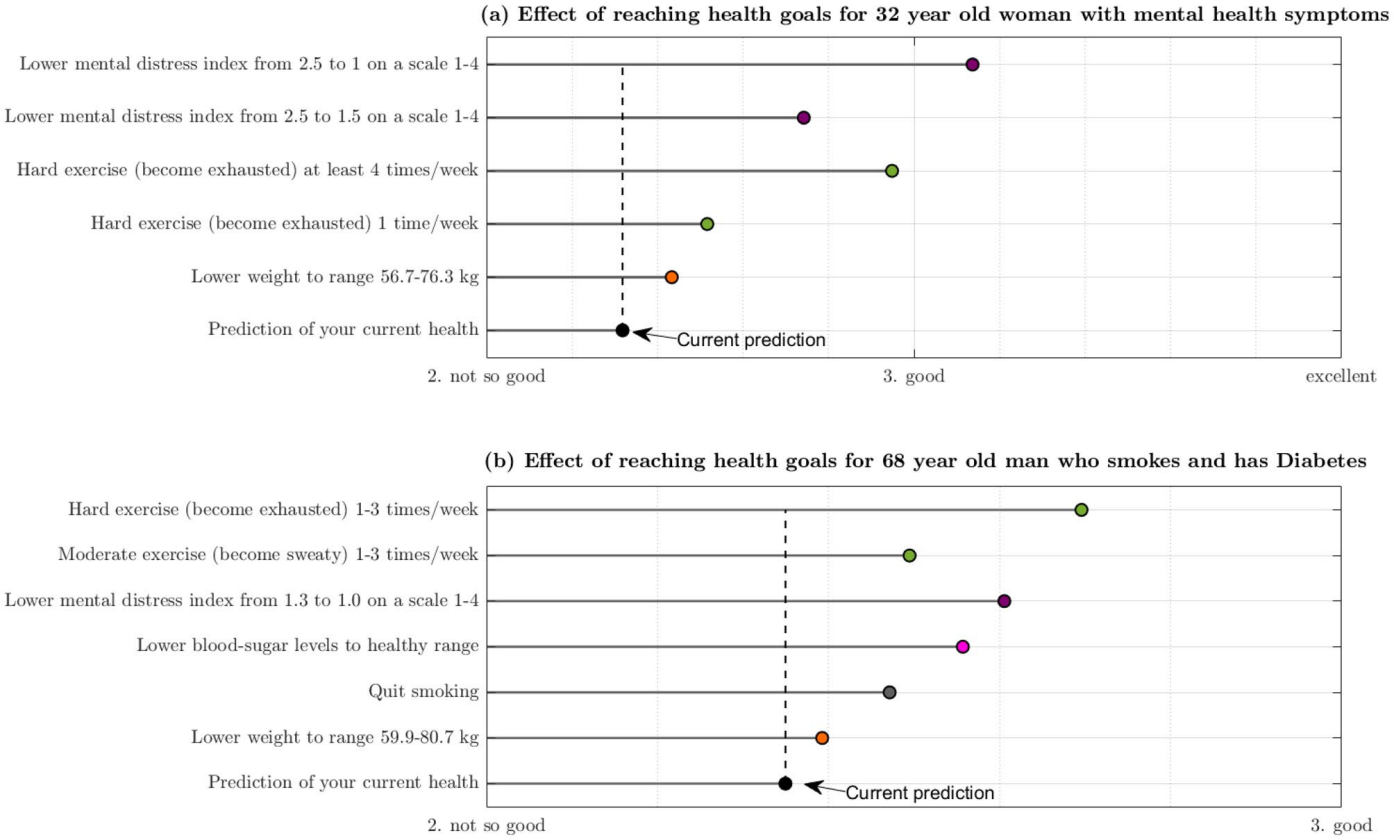
Examples of user tailored presentations based on the SRH-model using the lifestyle app. Predicted effect on SRH associated with achievement of each goal is plotted horizontally. The vertical dotted line shows the predicted SRH given the user's current status. Panel (**a**) corresponds to a fictional 68 year old man who exercises with mild intensity once per week, has no symptoms of mental distress aside from some slight sleep issues, smokes, has high blood sugar levels, and a BMI of 27.4 kg/m^2^. Panel (**b**) is a 32 year old woman who exercises with moderate intensity less than once per week, is experiencing severe psychological distress, and has a BMI of 27.0 kg/m^2^.

Figure 6 shows recommended health goals and their estimated effects on health for two fictitious app users. Figure 6a shows the app's suggestions for health targets for a 32 year old woman who experiences mental distress, exercises less than once per week, and has a BMI of 27.0 kg/m^2. The app suggests that mental health is the aspect of health that should be prioritised, followed by increasing the amount of PA. Reducing BMI from overweight to normal range is given lower priority. The expected SRH of someone with her profile is estimated to be intermediate between not so good and good, but improving her psychological wellbeing is predicted to increase her SRH to a level between good and excellent. Figure 6b shows automatically generated feedback for a 68 year old man who smokes, has HbA1c ≥ 6.5%, a BMI of 27.4 kg/m^2^, and reports exercising less than once per week with mild intensity. The app has accounted for his older age suggesting the optimal PA-schedule is exercise with high intensity 1–3 times per week (rather than ≥ 4 times/week), and by estimating the benefit of weight loss as smaller than it would be for someone < 65y.

## Discussion

We have fitted a statistical model of SRH and lifestyle factors to a large general cohort to model the relationship between modifiable health factors and SRH, and developed a proof-of-concept app that utilises this model as a knowledge base to recommend health goals to pursue and quantify their likely effects on health. The SRH-model provides a detailed, empirical description of the relationship between modifiable factors and SRH that considers multiple subgroups and interaction effects; by using the app to present a simple overview of the findings most relevant to each (fictitious) individual user, we have shown the potential of such apps to effectively integrate scientific models and communicate scientific health information. Our study illustrates how such apps can make health research more accessible to the general public through their ability to automatically individualise the presentation of such findings. Finally, we have highlighted a benefit of integrating multivariate epidemiological models into lifestyle apps; it allows the user to get a sense of the cost-to-reward ratios and impacts on health of a broad range of modifiable health factors which can help them prioritise the most impactful targets and develop more effective strategies.

### Model accuracy and validity

The model predicted SRH with decent accuracy, given its subjective nature. The variance in the random effects was high, indicating that we might be approaching an upper limit in terms of the accuracy with which SRH can be predicted. Consistent with this finding is that we found no indication of improved performance when we compared against more flexible machine learning algorithms (see Table S4), indicating that the main limiting factor for prediction accuracy may be lack of necessary information in the available data to predict the target, and not how well the models utilise that data. Since treating an ordinal endpoint as continuous can lead to effect miss-estimation in some circumstances^46^, we also compared the estimated effects against those of a Cumulative link model (see Fig. S2 in the supplementary materials), and found that this model agrees well in terms of effect sizes and confidence intervals, showing that the analysis is not unduly sensitive to choice of model, and that our treatment of SRH as a continuous variable did not substantially skew the effects that are of interest. Considering that both models lead to similar effect estimates and prediction accuracy, and that the regression model is the simpler of the two, we prefer to use it. However, it is worth noting that the assumption of conditional normality of SRH may be highly inaccurate when considering extremely ill individuals for which the SRH distribution may be skewed towards the lower end of the range, resulting in an asymmetric distribution.

### Comparison with previous literature on SRH

In the process of conducting a proof-of-concept study, we conducted a population study on the relationship between SRH and modifiable factors. We investigated a broad range of health related variables in a large and representative general cohort with high response rate, thus adding to the body of research on the connection between modifiable factors and SRH in a general population. We found that modifiable factors and healthy behaviours are strong determinants of SRH, which is consistent with previous findings on the relationship between SRH and healthy behaviours^47–56^. Our results agree with previous studies that PA is associated with better SRH^57^, and also that it seems to be a dose–response relationship (that is, a higher volume of PA is associated with a higher SRH); a result which has been observed for both objective and subjective measures of PA and fitness^48,55,57,58^. Our results are also consistent with previously reported associations between high BMI and poor SRH^51,56,59^ in high-income countries.

In a cross sectional health survey by Dalmasis et al. the authors found that poor sleep was the strongest modifiable predictor of poor SRH in a multivariate analysis that included smoking, PA, sleep, diet, and alcohol use^50^. Other studies we could find on sleep and SRH confirm that sleep is among the modifiable factors most strongly associated with SRH^49,52,54,59^. Initially, we included only the sleep score item from HSCL-10 as a covariate, and indeed found in that model that PA and issues with sleep were the strongest modifiable predictors of SRH (see Figure S4 for effects in the model that included sleep issues but not mental health, and Figure S5 for effects in model with both sleep issues and mental health). However, we decided to drop it from the model as we wanted to study the effect of mental health which correlated considerably with the sleep item.

We identified some notable discrepancies with previous results. Cullati et al. found in their multivariate study of the general Indian population that obesity and overweight were associated with better SRH when compared to normal BMI even after controlling for a broad range of demographic and socioeconomic confounders^60^. This discrepancy could be explained by India and Norway being low- and high-income countries respectively, and are culturally quite different. Another discrepancy we identified was a cross sectional health survey by Dalmasis et al. which found that smoking (smoker vs non-smoker) was as strongly associated with SRH as self-reported PA (vigorous or moderate PA vs low PA) in a multivariate analysis (OR for risk of poor vs good to excellent SRH: 1.34 and 1.38 respectively), whereas we found that PA was far more impactful. These discrepancies highlight the main potential drawback of using a subjective measure of health as an endpoint, namely that it could be strongly influenced by confounders such as socially conditioned beliefs about lifestyle and health that are hard to correct for, making it difficult to know what proportion of an association is due to causality.

### Advantage of apps as a means for communicating statistical models

To our knowledge, no current lifestyle apps use scientific models as a basis for recommending lifestyle changes or to quantify their benefits to health. Figure 6 provides an example of the kind of more individualised and precise feedback such an app could offer, which has been identified as an important factor in adherence^61^. It also illustrates how it allows comparison between (most of) the major domains of modifiable health factors, which can help a person invest their efforts where it likely matters the most. We also note how the presentation is kept simple despite the mathematical complexity of the underlying model, which highlights an advantage of using apps to present the research findings in general; it allows for simplification of complex information that, if conveyed verbally to a general audience, would form a complicated text with conditional “if–then” type sentences. This point is particularly relevant given the recent improvements in machine learning made possible by deep learning models and larger datasets, as these models can be significantly more accurate provided enough data, but lack the interpretability of traditional linear models. Our approach for presenting knowledge summarised by models does not require such interpretability however, as it uses the model only for predicting health differences between individual states.

Another advantage of our app-based method for conveying scientific results is that it allows the user to see and understand scientific results more directly, without relying on a “middle-man” for interpretation. Having access to a more raw and objective yet understandable form of research results could facilitate adherence, as seeing a less altered or interpreted form of results could make a person more inclined to believe in them. Apps incorporating our suggested approach could also facilitate patient-focused care, as the patient can see, without the ambiguity or bias that can be introduced in the translation of numerical results into words, the association between lifestyle change and health reward, making it easier for them to decide which investments offer the best effort-to-reward ratio. This would give the patient a more active role in improving their health, and doctor-patient communication could benefit from such technology, as they would have a simple-to-understand visual to refer to when discussing strategies for improving the patients' health.

### Comparison with clinical care

Using data-driven apps to guide patients on lifestyle change in pursuit of better health may result in prioritisations that are different from what is typically seen in a health care setting. For instance, if guidance was based on our app and SRH-model, mental health would likely be the most highly prioritised health factor for many individuals, as it was the strongest predictor of SRH by a substantial margin. The app would also tend to prioritise PA above both BMI and control of blood sugar levels. However, as in clinical care, the app takes into account the changing relationship between BMI and health with age, as it effectively places lower priority on weight loss after age 65. By including an interaction effect between age ≥ 65 years and vigorous PA approximately every day, the app also adjusts its feedback on PA levels on the basis of age, and conveys that extreme PA levels may be less advantageous in older age. Despite this negative interaction effect, vigorous PA was still strongly associated with high SRH in the age ≥ 65 cohort, illustrating another advantage of our suggested approach; it can more precisely communicate subtle interaction effects, resulting in a lower risk of miss-communication or exaggeration.

### Comparison between self-rated health and other health measures

In our analysis, we used SRH as a proxy measure of overall health. The relationship between SRH and lifestyle factors seems suitable for developing a model to improve general health and quality of life, as SRH, in addition to reflecting objective health status and mortality, also reflects subjective and positive components of health that go beyond mere absence of disease and disability. It provides a goal with high intuitive appeal that will appeal to many as it can be achieved in a shorter time frame than other often discussed health goals, such as risk-reduction for various age-related illnesses. The relationship between SRH and lifestyle factors can also be motivating due to how strongly SRH seems to be influenced by lifestyle factors. On the other hand, some individuals might find the subjective and seemingly flimsy nature of SRH unsatisfying, and it might therefore be beneficial to combine presentation of SRH-based results with findings that motivate the validity of SRH and its strong association to “harder” endpoints. Another argument for the utility of **SRH,** as a proxy for health used to study the effects of lifestyle on health, is that it is relatively easy (as it can be collected remotely via digital platforms) to collect large samples with sufficient representation of various subgroups to fit high resolution models that adjust their predictions based on the subgroup in question, which ultimately results in more individualised recommendations.

It is interesting to compare SRH more closely to longevity, the arguably most objective health related metric, as an endpoint to guide strategies and interventions for improving individual and public health through lifestyle and behaviour. Epidemiological studies consistently find a dose–response relationship between PA volume (intensity ⨉ frequency) and lifespan, but also that the biggest benefits are gained in the transition from a sedentary lifestyle to one that is moderately active (such as taking a brisk walk daily) with diminishing returns with increasing volumes^62–64^. We likewise observed a dose–response relationship, but we observed no evidence for diminishing returns. Indeed, our results suggest that even in quite physically active individuals there could still be considerable improvements to health and/or wellbeing in increasing PA levels further, even if benefits in mortality reduction might be close to exhausted at that point. Eriksen et al. similarly found a strong relationship between most vigorous level of exercise and fitness and high likelihood of good SRH (OR: 12.2; moderate/vigorous PA and high cardiorespiratory fitness vs low PA levels and cardiorespiratory fitness) in a cohort of healthy adults, with no indication of diminishing returns with greater PA volume. Thus, if SRH was used as an endpoint for guiding lifestyle changes, the literature would tend to emphasise not just exercising, but exercising vigorously, in contrast to weighting the importance of lifestyle factors based on longevity as an endpoint. Conceivably, the difference in the dose–response relationships is due to SRH reflecting short-term changes in mood and well-being that are not captured in longevity studies. On the other hand, analysing longevity may detect long term consequences of behaviours that are poorly captured by subjective experiences. For example, smoking had a surprisingly weak association with SRH that did not seem to be proportional to its well known health consequences. A SRH-based model of health could therefore supplement a longevity-based model to create a more complete picture that considers both long term and short term health outcomes. Another reason why a SRH model in isolation might not be suitable for representing the harm of smoking, is that the increased mortality risk associated with smoking could substantially bias its effect in SRH-models since the most severely affected individuals are less likely to be able to participate in the study. To get a more complete understanding of the benefits of each health-related behaviour, we recommend that lifestyle apps based on statistical models should incorporate models for different measures of health, such as mortality, specific diseases (such as cardiovascular diseases, obesity or diabetes type 2), and mental health.

## Limitations

### Poor sleep is always attributed to mental distress

We modelled HSCL-10 as a continuous variable that does not reflect which items are contributing to the score, and the effect of this is that the app will indicate significant potential improvement in SRH via reduction of mental health symptoms based on a high value on the insomnia item alone. It might therefore be appropriate to require a criteria to be met demanding contribution from multiple items, such as HSCL-10 exceeding some minimum threshold or a minimum number of HSCL-10 items indicating mental distress, before triggering the app's recommendation to work on mental health and attributing sleep issues to poor mental health.

### Subjectivity in choice of goals to present

To keep the presentation simple and accessible, especially to individuals with reduced abilities to process large amounts of presented information, we chose to have the app present only a subset of possible suggestions for PA-related goals depending on the user case. These choices were admittedly somewhat arbitrary, and ideally such choices should be based on relevant fields of research such as behavioural science. An alternative approach would be to add a button for showing all the possible goals and changes, as some people might find it interesting to get a more comprehensive view and a more complete understanding of how health connects to lifestyle factors. It could perhaps also be interesting to have a button that reveals negative changes, so that a person can see not just where there is room for improvement, but also be motivated by the benefit of the healthy behaviours they are already maintaining.

### Bias in selfreported metrics

A well known issue with self-report data is self-report bias, typically in the form of overestimation of healthy behaviours. For SRH, health optimism has been shown to predict lower mortality in people with poor objective health^65^, and some authors have suggested that this particular “bias” could conceivably play a causal role in the development of future health status^36^, in which case health optimism would itself be a goal to strive for. Report bias or reporting behaviour can however skew apparent causal connections when the bias in reporting the behaviour correlates with the bias in reporting subjective health. Pushing this idea to its extreme, the association between SRH and a self-reported lifestyle variable would represent only an underlying “optimism” in how the individual considers his/her own health, in which case adopting a healthy behaviour without adopting the health optimism would not produce the changes in SRH indicated by the analysis. We can not rule out such a tendency for report biases to correlate as a source of error and effect inflation. We note however, that if biases are reasonably consistent across individuals (for instance, if everyone tends to overestimate PA levels, and not just “health optimists”), then the model effects would still be useful for comparison purposes since the biases would cancel out in the comparison. Also, if the biases are consistent across time, an analysis that considers changes within individual trajectories can potentially discover such biases, and since mixed effect models can consider within subject changes they can ameliorate this issue. However, the potential for correcting for this effect depends on the number of datapoints per participant, and it is unclear if collecting 2 data points per person for a subset of participants makes a substantial difference in this regard. An improvement would therefore be to collect a greater number of consecutive data points per person.

### Challenges of interpretation and inferring causality

Perhaps the biggest limitation of this study is that the data is observational, and therefore we can not infer causality. Interpreting associations and deciding which variables to include is also made more challenging by complicated relationships between many of the model variables, with some acting as both confounders and mediators for other covariates, and some covarying considerably. For example, PA's positive effect on health is partly mediated through its ability to facilitate long-term maintenance of weight loss^66^, and thus BMI can act as a mediator of the positive effect of PA on SRH (Fig. S1 in the supplementary materials shows that the effect of PA drops when BMI is controlled for). Conversely, being overweight can cause an individual to be less motivated to exercise, and therefore BMI can also be viewed as a confounder in this context. Consequently, one can argue for either inclusion or exclusion of e.g. BMI depending on prior beliefs about the underlying relationship between the variables. A similar discussion can be had for PA and comorbid disease burden^5^, PA and blood-sugar levels, and other combinations of model variables.

Another potential source of effect over- or under-estimation is reverse causality. A relationship where causality likely goes in both directions is that between self-reported mental health SRH. However, adjusting for comorbid disease provides some degree of confidence that HSCL-10 is not merely a reflection of poor health; if the causal relationship was mainly that poor health leads to poor mental health we would expect the impact of HSCL-10 to become small when controlling for objective health, which was not the case.

### Variables do not change in isolation

The app presents the importance to health of a modifiable factor in terms of the effect of changing it under the assumption that all other variables are held fixed. This can be a highly artificial assumption, and a presentation that does not properly account for this fact risks misleading people into thinking that a presented value represents the sum total of direct and indirect effects associated with reaching a health goal, and thus underestimating its true impact.

Sleep and mental health highlight especially well the complications arising from complex interdependence between model variables in conjunction with benefits being presented in terms of isolated changes. If both variables are included in the model, then the interpretation of the effect of resolving sleep issues is the expected improvement to SRH given that all other covariates, including mental health, do not change. This is highly unrealistic for many individuals to whom improving sleep would likely improve their mental health considerably, thereby underestimating the true ramifications that improved sleep would have for those individuals. The simplest solution (which we opted for in this study) is to exclude one of the variables, sleep in this case, from the model as an explicit variable. However, this solution is unsatisfying since sleep issues can exist independently from mental health issues, and for individuals whose sleep is not rooted in psychological issues it is confusing, misleading, and inaccurate to attribute their sleep issues to poor mental health.

A strategy is to collect the data necessary to better separate sleep and mental health issues, for example by using sleep questionnaires to classify sleep issues; a more detailed description could be used to form a modified version of HSCL-10 where sleep issues contribute to the HSCL-10 rating only when they cannot be attributed to poor sleep-related habits, thus reducing collinearity and allowing sleep to contribute as an independent predictor of SRH. An alternative solution that can be achieved without collecting additional data is to consider ways to aggregate the HSCL-10 items into a smaller set of summary variables such as anxiety, depression, and sleep issues.

### How to overcome uncertainties concerning causal interpretations

Although the model alone can (under the idealised assumption) provide information on what goals would make good subjective health likely if achieved, it does not give detailed information on how to achieve those changes nor how difficult they are to achieve. As we have discussed, the causal impact of reaching certain goals might also be underestimated if they exert some of their effects through other variables in the model. For these reasons, it is natural to consider incorporation of other more specialised sources of information to enter the app's flow of operations. For instance, if the user selects mental health as a goal to pursue, this could trigger a cascade of successively more specialised queries that aim to map out the user's mental state in greater detail and help them create a more detailed strategy for achieving that goal. This strategy would then likely involve improving on other factors included in the model, such as increasing physical activity to improve sleep or mental distress, based on previous research which has demonstrated (causally, in the ideal scenario) this benefit. In this way, variables that are underestimated due to some of their indirect effects being attributed to other effect-mediating variables, can have their importance properly evaluated in subsequent strategization steps. With this scheme, the role of the epidemiological model is not to inform the user on the exact sequence of actionable goals to aim for, nor the true causal impact on SRH of achieving them, but rather to provide a comprehensive overview of the major levers of health and help them outline suitable long term goals. The epidemiological model would then serve primarily as a tool that provides an initial “nudge” in the direction of relevant literature and resources, which is appropriate given the limitations we have discussed.

Another way to overcome the issues of presenting the benefits of isolated changes, is to communicate to the user how to correctly interpret the presented results, and inform them that, in reality, positive lifestyle changes rarely occur in isolation but are likely to have positive knock-on effects. A simple example of integrating this idea into the app design is to allow the user to query the model for the joint effect of reaching multiple goals. Another suggestion for further improvement is to have the app suggest synergistic combinations based on relevant literature, such as simultaneously aiming to improve sleep (sleep issues is included HSCL-10) and getting more frequent exercise^67^.

### The model cannot show cumulative effects over time

The model and app cannot provide information on the cumulative benefit or harm of maintaining behaviours or states across various lengths of time, and does not specify the time of adherence that corresponds to the stated effects. Measures of PA, blood sugar control, BMI, and mental health in this study provide only snapshots of peoples current situations at two time points. Therefore, the SRH associated with a certain reported behaviour, such exercising 2–3 times per week, will represent an average across various levels of adherence to that behaviour, and the estimated effect may therefore not represent the benefit of long term adherence. This again stresses the importance of educating the user on the correct interpretation and limitations of the presented information, as well as supplementing it with scientific results that motivates adherence by illustrating the cumulative nature of various lifestyle changes.

### Classification of variables into modifiable and non-modifiable

Some variables are in a grey area with regards to whether or not they are modifiable. We originally considered treating “support from friends” as a modifiable variable, since personal relationships can in some cases be improved through controllable factors like time invested. However, there are also factors involving complex interpersonal dynamics that are not under the direct control of the individual. It would be interesting to consider how the social domain of health could be appropriately integrated in the kind of lifestyle app we propose, but doing so here goes beyond the scope of this study and the variables available for analysis.

### Not all aspects of lifestyle are covered

We have not included as independent predictors the full range of modifiable lifestyle factors relevant to health. Most notably, diet and sleep are not (explicitly) included. Numerous studies have found that diet is associated with SRH after adjusting for other lifestyle factors^49–51,54^, and should therefore ideally have been included in this study.

### Difficulty evaluating certain interaction effects due to small overlaps

Pairwise interactions between certain conditions were hard to evaluate robustly due to their intersection containing few participants. Most notably, interaction effects between underweight BMI and age ≥ 65y or high PA levels and age ≥ 65y were estimated with high uncertainty due to few individuals meeting both conditions. Therefore, we cannot claim confidently that the model accurately reflects how age modifies the effect of these particular variables over time.

### Results are speculative

This is a proof-of-concept study where we argue for the advantages of incorporating scientific models into lifestyle apps, but we do not actually test the claim that implementation of such tools leads to improved adherence to healthy habits. Randomised control trials are needed to validate these claims.

## Conclusions

A digital educational tool for presenting scientific findings that utilises user data to adjust the presentation can potentially convey research findings more effectively than standard guidelines. They can visualise the information from a point of reference that is tailored to the user, simplify the presentation by showing only relevant findings, and facilitate engagement and understanding by allowing the user to interact with the information, and convey health information in a way that is more easily translated into actionable steps. SRH was found to be strongly associated with lifestyle factors. The most predictive modifiable health risk factors were symptoms of mental health, physical activity levels, and BMI. PA frequency and intensity were found to interact positively on SRH, suggesting that PA that high volume is particularly important for good SRH.

### Supplementary Information

Supplementary Information 1.

## Data Availability

The data that support the findings of this study are available from The Tromsø Study (https://uit.no/research/tromsostudy) but restrictions apply to the availability of these data, which were used under licence for the current study, and so are not publicly available. Data are however available from the authors upon reasonable request and with permission of The Tromsø Study. Descriptions of the questionnaire variables used in this study can be found at http://tromsoundersokelsen.uit.no/tromso/. The code is open sourced using the The GNU Affero General Public License. It is available at https://github.com/uit-hdl/health-diary-app.

## Abbreviations

SRH: Self-rated health
PA: Physical activity
IAPHI: Individually adjusted presentation of health information
BMI: Body mass index
HSCL: Hopkins symptoms checklist
HII: Health impact index
