# Supplementary material for: An individually adjusted approach for communicating epidemiological results on health and lifestyle to patients
Source: Sci Rep. 2024 Feb 8;14:3199. doi: 10.1038/s41598-024-53275-x (PMC10853548; doi:10.1038/s41598-024-53275-x)
Supplement: Supplementary file 1 — Supplementary Information 1. [file 41598_2024_53275_MOESM1_ESM.docx]

Supplementary materials

**Table S1.** Number of participants with data on all variables in the SRH-model.

| **Cohort** | **N.o. survey invitees who responded to email questionnaire** | **N.o. participants with complete data for analysis [% of respondents]** |
| --- | --- | --- |
| Tromsø6 | 12 981 | 10 604 (81.7%) |
| Tromsø7 | 21 083 | 18 441 (87.5%) |
| Tromsø6 and Tromsø7 | 8 906 | 6 710 (75.3%) |
| Tromsø6 or Tromsø7 | 25 158 | 22 335 (88.8%) |

**Table S2.** Comparison of cohort characteristics between individuals who participated in Tromsø6 but did not return in Tromsø7 and those that participated in both waves.

|  | **SRH mean** | **Age mean** | **BMI mean** | **HSCL mean** | **HII mean** | **HII≥3 [%]** | **Female [%]** |
| --- | --- | --- | --- | --- | --- | --- | --- |
| Individuals who participated in both surveys, Tromsø6 survey | 2.83 | 55.81 | 26.9 | 1.27 | 0.68 | 8.6% | 53.6% |
| Individuals who participated only in Tromsø6 | 2.57 | 61.25 | 27.0 | 1.34 | 1.00 | 16.3% | 52.8% |

Note: Values are from the Tromsø6 survey. SRH=Self-rated health. HII=Health impact index. HSCL=Hopkins symptoms checklist.

### Investigating interaction effects

To investigate if the effects of physical activity levels or body mass index were influenced by sex or comorbidity status, we included interaction effects in the full model, and evaluated the p-value of the corresponding interaction term. In our case, this is a binary term “A:B” that equals 1 if both conditions are true and 0 otherwise, which effectively amounts to studying the mean SRH for the subgroup that is the intersection of A and B. If the p-value was high (>0.05) we concluded that the effect was the same in each subgroup. No significant modification of the effect of BMI or physical activity levels were observed, as the corresponding p-values were all >0.1.

**Table S3.** Statistical significance of interaction effects for comorbidity status and sex.

| **Interaction** | **Coefficient estimate** | **p-value** | **N participants in intersection, Tromsø6** | **N participants in intersection, Tromsø7** |
| --- | --- | --- | --- | --- |
| Health Impact index (comorbid disease burden) | | | | |
| HII≥2 : “hard PA ≥ 4 times/week” | -0.0036 | 0.9470 | 14 | 32 |
| HII≥3 : “hard PA ≥ 2-3 times/week” | 0.0683 | 0.6286 | 44 | 127 |
| HII≥3 : “PA < 1 time/week” | 0.0242 | 0.6287 | 993 | 867 |
| HII≥3 : “normal BMI” | -0.0752 | 0.1065 | 405 | 272 |
| HII≥3 : “obese BMI” | 0.0708 | 0.1124 | 406 | 352 |
| Sex | | | | |
| Male : “hard PA ≥ 4 times/week” | -0.0224 | 0.8525 | 49 | 108 |
| Male : “hard PA ≥ 2-3 times/week” | 0.0289 | 0.6894 | 146 | 312 |
| Male : “PA < 1 time/week” | -0.0028 | 0.9209 | 4343 | 7985 |
| Male : “normal BMI” | -0.0336 | 0.1535 | 1697 | 1697 |
| Male : “obese BMI” | 0.0085 | 0.7425 | 1244 | 2509 |

Note: The two columns furthest to the right show the number of participants that satisfied both conditions, such as “is male and “exercises < 1 time/week”, in each survey. This information was included to get a sense of whether the lack of significance was due absence of an effect or an underpowered sample. PA=Physical activity. HII=Health impact index.

### Comparing models

An advantage of conveying health research in terms of expected effects or benefits associated with various changes is that a complex model can be reduced to a single number at the individual's request, and thus removes the modelling constraint that the model has to be verbally communicable. It is therefore interesting to consider what performance could be gained by shifting focus to pure performance optimisation, and utilising more complex and flexible models and machine learning techniques. To this end, we compare the performance of the linear mixed effects model (the LMM) in 5-fold cross validation against two machine learning algorithms that have demonstrated high performance on structured data: Explainable boosting machines (EBM) and Extreme Gradient Boosting^[[1]](#footnote-1)^. XGboost is a form of decision tree ensemble model that combines a number of weaker models to produce a single strong one. EBM is a kind of generalised additive model that also incorporates pairwise interaction terms, and achieves similar performance to state-of-the-art blackbox models whilst being interpretable. These models were given the raw variables as inputs, without performing any thresholding or discretization. We also compare against a Cumulative link model (CLM); an ordinal regression model with random effects^[[2]](#footnote-2)^ which differs from the assumptions of the regression model in that it does not assume equidistance between the ordinal categories of the independent variable. The comparison metrics are AUC for predicting poor and excellent SRH respectively.

Confidence intervals are computed using standard statistical methods where we assume that the 5 cross validation estimates are independently and normally distributed. All models are fitted to the joint Tromsø6 + Tromsø7 dataset described in the paper.

In the main model, we use HSCL-10 to represent an aggregate representation of 10 questionnaire items that reflect mental distress and sleep issues. From the point of view of building an accurate predictive model, averaging across these values may not be the optimal way to aggregate them, and we test letting each model find the optimal aggregation method by entering them into the model as separate features. Dropping all rows that have 1 or more missing HSCL-10 would lead to dropping a large portion of the dataset, so we instead drop the rows with more than 3 missing values, and for the remaining rows we fill in the missing values with the mean for each respective item.

**Table S4.** Cross validation comparison of mixed effects regression models against

| **Model** | **AUC [poor SHR]** | **AUC [excellent SHR]** |
| --- | --- | --- |
| M0: same variables the main model of the paper | | |
| Mixed effects linear regression | 0.8036 (0.795, 0.812) | 0.7791 (0.774, 0.784) |
| Mixed effects ordinal regression | 0.8022 (0.794, 0.811) | 0.779 (0.775, 0.783) |
| XGboost regression | 0.8004 (0.789, 0.812) | 0.7804 (0.778, 0.783) |
| EBM regression | 0.8058 (0.795, 0.816) | 0.7805 (0.776, 0.785) |
| M1: same as M0, but leaving the HSCL-10 variables as separate predictors | | |
| Mixed effects linear regression | 0.8222 (0.812, 0.832) | 0.7802 (0.77, 0.79) |
| Mixed effects ordinal regression | 0.8217 (0.812, 0.831) | 0.7776 (0.766, 0.789) |
| XGboost regression | 0.8205 (0.81, 0.831) | 0.7802 (0.769, 0.791) |
| EBM regression | 0.8215 (0.811, 0.832) | 0.7814 (0.771, 0.792) |

There is no significant difference in performance between the 4 models. However, there is a slight but significant increase in performance for the models that take the HSLC-10 items as input directly instead of their average as single aggregate representation. Only the ability to discern poor SRH improved however, whereas discerning excellent SRH did not improve. In general, the negligible performance differences between the models indicates that the main factor restricting prediction performance is lack of predictive power in the data available, and not how the models utilise that data.

We can think of 3 factors that might limit prediction accuracy: 1. There are important variables not in our dataset that would improve prediction accuracy substantially if included, 2. Some of the predictors may have considerable measurement noise, such as self reported PA, which could cause loss of information in the predictors, 3. SRH is inherently somewhat unpredictable from traditional measurements due to dependence on subjective factors that are hard to measure and/or fluctuate on a shorter time scale, such as mood at the time of answering. Explanation 1 is plausible, in particular because we have not included dietary information. Explanation 2 seems less likely; the error/bias in self-reported PA might even be predictive of SRH, so it is not obvious that more objective measures would improve accuracy. We suspect that explanation 3 is the most important limiting factor. In any case, the important measure of accuracy is how accurately the model parameters portray the causal effects and the relative importance of the modifiable factors, and this portrayal can be accurate even if accuracy with which SRH is predicted is limited.

### Paradoxical PA findings: more frequent exercise associated with lower SRH

Curiously, those who reported mild PA only once per week had slightly higher average SRH than those who reported mild PA 2-3 times per week in the fully adjusted model. Indeed, we found a higher prevalence of participants who had a history of stroke, heart attack, or HII≥2 in the “mild PA 1/week” group than in the “mild PA 2-3 times/week” group. In the Tromsø6 dataset, this subgroup of moderately to seriously ill participants constituted **35.9%** (95% CI: 33.6-38.2) of the “mild PA 2-3 times/week” group, but only **30%** (CI: 27.4-32.6) of the “mild PA 1/week” group (p=0.0008). Similarly, the corresponding values in the Tromsø7 dataset were **33%** (31.2-34.9%) for the “mild PA 2-3 times/week” group and **30.1%** (27.8-32.5%) for the “mild PA 1/week” group (p=0.056). Thus, it appears plausible that participants who engage in mild but frequent exercise sessions in response to illnesses or perceived poor health might confound the relationship between mild-intensity PA and SRH. Ideally, such a confounding effect would be corrected by including the HII. However, HII may not describe the comorbidity status of the participants in sufficient detail to adequately correct for the aforementioned confounding effect since the questionnaire items do not discern between severity nor timing of events and conditions, and the illnesses considered are not exhaustive. Consequently, this study's results on the relationship between mild PA and SRH may not be reliable, and further investigation on the relationship between mild PA and SRH is warranted.

### Sensitivity analysis

#### Justification for treating SRH as a continuous variable

For the sake of simplicity, we modelled SRH as a continuous variable, but another common (and sometimes more appropriate) modelling approach for ordinal data is to use a CLM. The CLM assumes that ordered categories represent a discretization of a normally distributed latent variable whose mean depends linearly on the model covariates. Specifically, it assumes that the ordinal categories are obtained by dividing the number line into segments with a number (n categories - 1) of thresholds, and assigning categorical values based on which bin each latent variable falls into. Depending on the distribution of the thresholds and the nature of the latent variable, treating SRH as a continuous variable may lead to incorrect conclusions and miss-estimations. It is important to investigate whether this modelling choice makes a difference to the inferences we draw from the analysis, and if our assumption of equidistant SRH levels is reasonably correct.

We test robustness to modelling choice by fitting both models to the joint Tromsø 6 and 7 dataset described in the article. First we compare the coefficients of the two models. Both models consider effects on a normally distributed variable that represents SRH, so this comparison makes sense provided that we account for differences in scale. We normalise the coefficients of each model by dividing by the absolute value of the largest coefficient of each fit, thus allowing us to compare the relative importance that each model assigns to different variables and effects. The results are shown in **Figure S2** which offers a side-by-side comparison of each pair of estimates. Note we have converted HSCL-10 into a categorical variable for easier comparison. The relative importance and prioritisation of the variables, the width of the confidence intervals, and the significance of the estimated effects are reasonably similar between the two models. We therefore conclude that inferences and conclusions are not unduly sensitive to choice of model, and that modelling SRH as a continuous and equidistantly spaced variable is a valid approximation.

As an additional test of the assumption of equidistance, we compare the spacing of the 3 thresholds that separates the sections of the number line corresponding to the 4 SRH categories (these are estimated as parameters when fitting the model). Interval 2 and 3 (corresponding to the SRH=2 and SRH=3 respectively) are of finite lengths and can be compared. We obtain the following thresholds separating the SRH categories: 7.11, 4.37, and 1.16. The widths of interval 2 and 3 are 2.74 (95% CI: 2.68, 2.8) and 3.22, (3.17, 3.26) respectively. Interval 1 is 15% shorter than interval 2, indicating that the assumption of equidistance is reasonable, albeit not perfect.

Finally, we compare the goodness of fit for the two models using the Bayesian information criterion (BIC). The BIC is a method of comparing non-nested models that rewards high likelihood of the observed data whilst penalising high model complexity, with lower BIC indicating a more preferable model. The BIC for the LMM was 58 913 and the BIC for the CLM was 62 497, which supports using the LMM model.

#### Choice of variables to include in the model

**Figure S1** displays the results of the sensitivity analysis examining how the impact of physical activity (PA) and BMI on self-rated health (SRH) varies depending on the variables included as controls. **Figure 3a** shows the adjusted difference in SRH for those who reported hard PA≥4 times/week compared to the sedentary group (mild PA<1 time/week) across various models. **Figure 3b** shows the outcome of the analysis for normal BMI compared to obese BMI in models that adjust for Hypertension, Hypertension+HbA1c, and Hypertension+HbA1c+HII. Comparing the adjusted impact of vigorous PA in the base model vs the model that controls for mental health and comorbid disease burden, the impact drops from 0.745 (0.66-0.83) to 0.646 (0.57-0.72), which is a reduction in estimated impact by 13.3%. Comparing the adjusted impact of BMI in the base model (M0) vs the model that controls for mental health and comorbid disease burden (M3), the effect drops from 0.315 (0.29-0.34) to 0.226 (0.23-0.27); a reduction in the estimated impact of BMI by 20.6%.


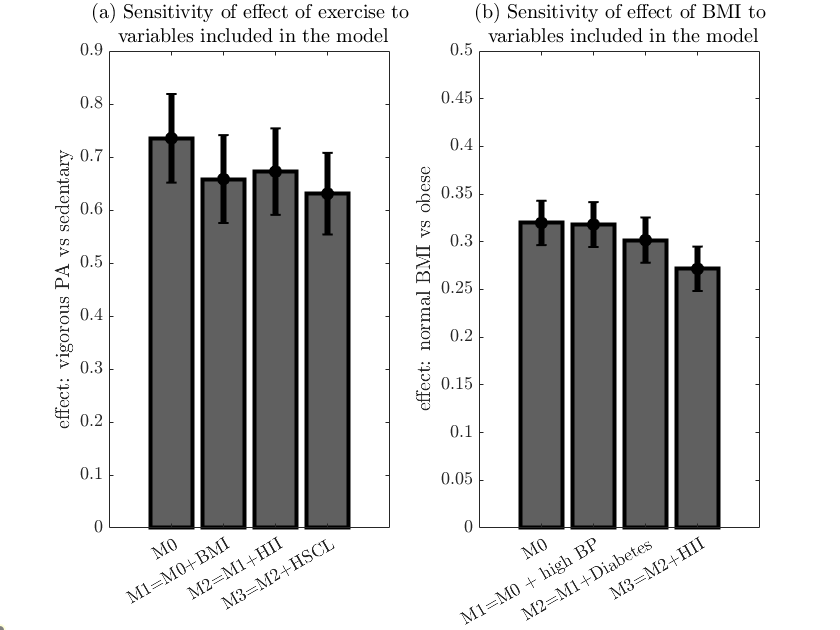


**Figure S1:** Sensitivity analysis for examining how the choice of variables included as controls influences the estimated impact on SRH of PA and BMI. The models are nested, and control for one additional variable going left to right. The baseline model M0 adjusts for age, sex, education, and smoking. Panel **(a)** shows the adjusted difference in mean SRH between those who were sedentary (mild PA<1 time/week) and those who reported vigorous exercise (hard PA≥4 times/week) using each resective model to estimate this effect. Panel **(b)** shows the adjusted difference in mean SRH between normal and obese BMI for the respective models. HII=Health impact index. HSCL=Hopkins symptoms checklist. PA=Physical activity. SRH=self-rated health.


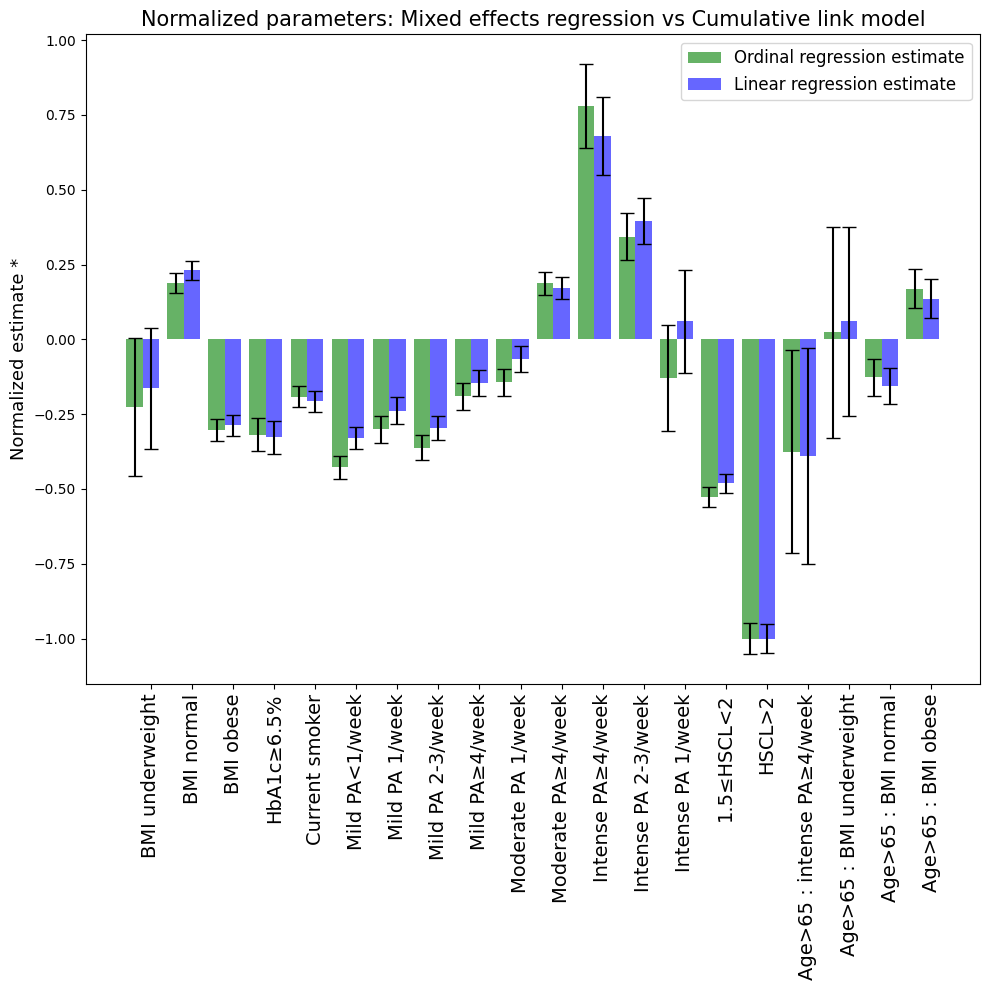


**Figure S2:** Parameter estimates from fitting a Cumulative Link model (green) and mixed effects regression model (blue) with 95% confidence intervals. The parameters have been normalised by dividing by the magnitude of the largest estimated effect (the parameter of HSCL-10>2 in each case) for each model respectively. HSCL=Hopkins symptoms checklist.


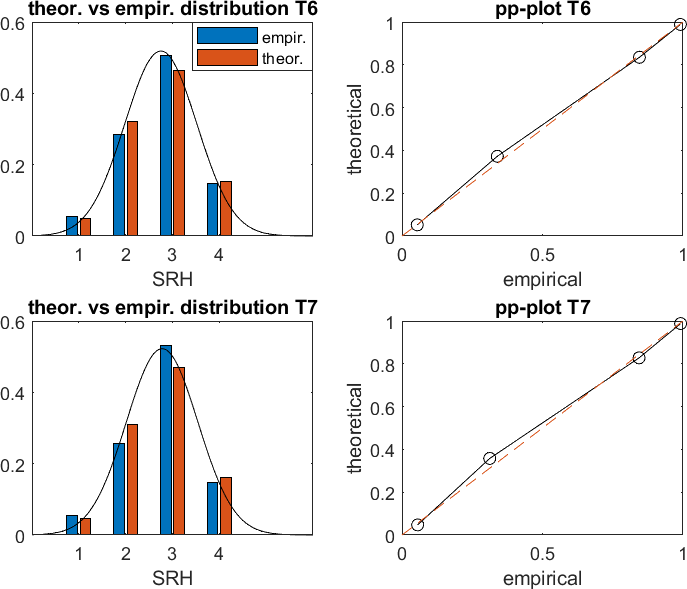


**Figure S3:** Theoretical vs empirical normal distribution of SRH in Tromsø 6 and Tromsø 7. The theoretical probabilities for each of the 4 SRH levels were predicted by rounding the continuous output of the SRH-model to the nearest integer in the range 1-4. The right-hand figures are probability-probability plots showing the empirical vs theoretical rates of SRH≤i for i=1, 2, 3 and 4.


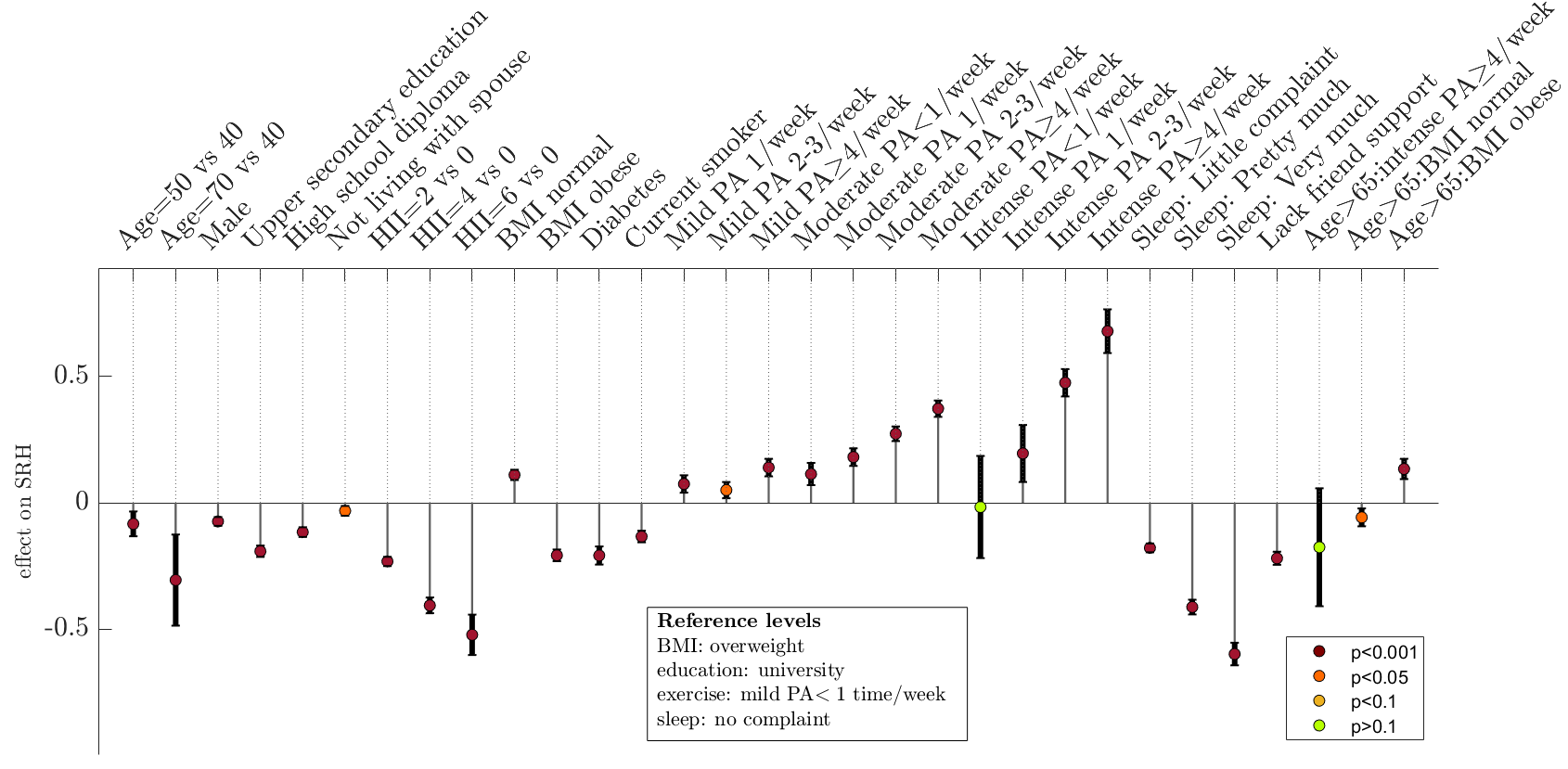


**Figure S4: Effects in the model which included sleep issues but not mental health.**


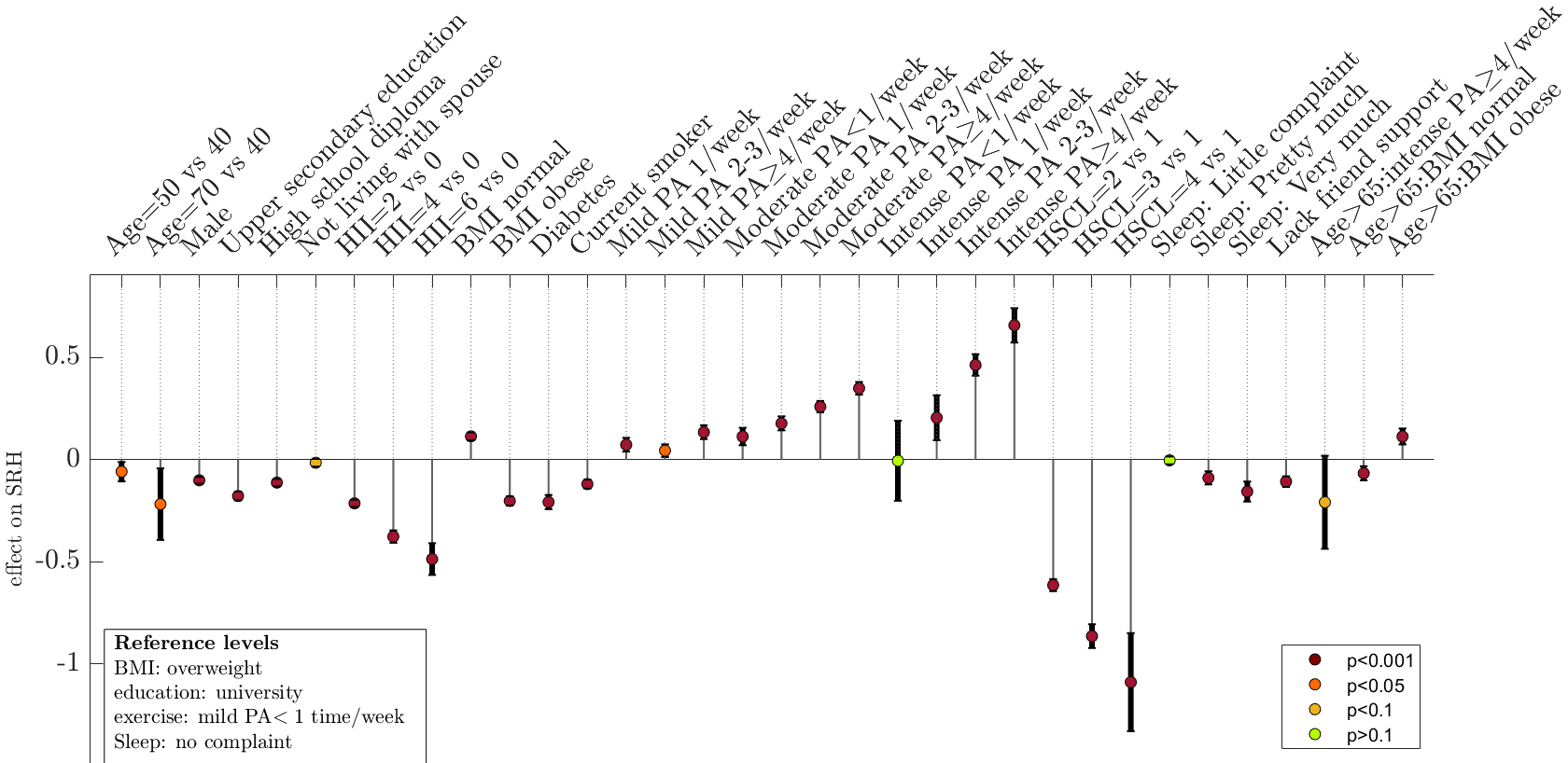


**Figure S5:**  **Effects in the model that includes both sleep issues and mental health.** This model included the sleep issue item in HSCL-10 as well as mental health as measured by HSCL-10.

1. To fit these models we used the Python libraries xgboost and ExplainableBoostingRegressor. For XGboost we used the settings: learning_rate=0.1, max_depth=5, n_estimators=4000, subsample=0.6, colsample_bytree: 0.6, early_stopping_rounds: 10, eval_metric= "rmse", objective_params="reg:linear". Tuning the parameters had no significant effect on performance. [↑](#footnote-ref-1)
2. For the Ordinal regression mixed effects model we used the logit link function, and the OrdinalGEE Python library. [↑](#footnote-ref-2)
